# ImageJ2: ImageJ for the next generation of scientific image data

**DOI:** 10.1186/s12859-017-1934-z

**Published:** 2017-11-29

**Authors:** Curtis T. Rueden, Johannes Schindelin, Mark C. Hiner, Barry E. DeZonia, Alison E. Walter, Ellen T. Arena, Kevin W. Eliceiri

**Affiliations:** 1 Laboratory for Optical and Computational Instrumentation, University of Wisconsin at Madison, Madison, Wisconsin USA; 2 Morgridge Institute for Research, Madison, Wisconsin USA

**Keywords:** ImageJ, ImageJ2, Image processing, N-dimensional, Interoperability, Extensibility, Reproducibility, Open source, Open development

## Abstract

**Background:**

ImageJ is an image analysis program extensively used in the biological sciences and beyond. Due to its ease of use, recordable macro language, and extensible plug-in architecture, ImageJ enjoys contributions from non-programmers, amateur programmers, and professional developers alike. Enabling such a diversity of contributors has resulted in a large community that spans the biological and physical sciences. However, a rapidly growing user base, diverging plugin suites, and technical limitations have revealed a clear need for a concerted software engineering effort to support emerging imaging paradigms, to ensure the software’s ability to handle the requirements of modern science.

**Results:**

We rewrote the entire ImageJ codebase, engineering a redesigned plugin mechanism intended to facilitate extensibility at every level, with the goal of creating a more powerful tool that continues to serve the existing community while addressing a wider range of scientific requirements. This next-generation ImageJ, called “ImageJ2” in places where the distinction matters, provides a host of new functionality. It separates concerns, fully decoupling the data model from the user interface. It emphasizes integration with external applications to maximize interoperability. Its robust new plugin framework allows everything from image formats, to scripting languages, to visualization to be extended by the community. The redesigned data model supports arbitrarily large, N-dimensional datasets, which are increasingly common in modern image acquisition. Despite the scope of these changes, backwards compatibility is maintained such that this new functionality can be seamlessly integrated with the classic ImageJ interface, allowing users and developers to migrate to these new methods at their own pace.

**Conclusions:**

Scientific imaging benefits from open-source programs that advance new method development and deployment to a diverse audience. ImageJ has continuously evolved with this idea in mind; however, new and emerging scientific requirements have posed corresponding challenges for ImageJ’s development. The described improvements provide a framework engineered for flexibility, intended to support these requirements as well as accommodate future needs. Future efforts will focus on implementing new algorithms in this framework and expanding collaborations with other popular scientific software suites.

**Electronic supplementary material:**

The online version of this article (doi:10.1186/s12859-017-1934-z) contains supplementary material, which is available to authorized users.

## Background

ImageJ [1] is a powerful, oft-referenced platform for image processing, developed by Wayne Rasband at the National Institutes of Health (NIH). Since its initial release in 1997, ImageJ has proven paramount in many scientific endeavors and projects, particularly those within the life sciences [2]. Over the past twenty years, the program has evolved far beyond its originally intended scope. After such an extended period of sustained growth, any software project benefits from a subsequent period of scrutiny and refactoring; ImageJ is no exception. Such restructuring helps the program to remain accessible to newcomers, powerful enough for experts, and relevant to the demands of its ever-growing community. As such, we have developed ImageJ2: a total redesign of the previous incarnation (hereafter “ImageJ 1.x”), which builds on the original’s successful qualities while improving its core architecture to encompass the scientific demands of the decades to come. Key motivations for the development of ImageJ2 include:

1. **Supporting the next generation of image data.** Over time, the infrastructure of image acquisition has grown in sophistication and complexity. For example, in the field of microscopy we were once limited to single image planes. However, with modern techniques we can record much more information: physical location in time and space (X, Y, Z, time), lifetime histograms across a range of spectral emission channels, polarization state of light, phase and frequency, angles of rotation (e.g., in light sheet fluorescence microscopy), and high-throughput screens, just to name a few. The ImageJ infrastructure needed improvement to work effectively with these new modes of image data.
2. **Enabling new software collaborations.** The field of software engineering has seen an explosion of available development tools and infrastructure, and it is no longer realistic to expect a single standalone application to remain universally relevant. We wanted to improve ImageJ’s modularity to facilitate its use as a software library, the creation of additional user interfaces, and integration and interoperability with external software suites.
3. **Broadening the ImageJ community.** Though initially developed for the life sciences, ImageJ is used in various other scientific disciplines as well. It has the potential to be a powerful tool for any field that benefits from image visualization, processing, and analysis: earth sciences, astronomy, fluid dynamics, computer vision, signal processing, etc. We wanted to enhance ImageJ’s impact in the greater scientific community by adopting software engineering best practices, generalizing the codebase, and providing unified, comprehensive, consistently structured, community-editable online resources.

From these motivations emerge the six pillars of the ImageJ2 mission statement:

- **Design** the next generation of ImageJ, driven by the needs of the community.
- **Collaborate** across organizations, fostering open development through sharing and reuse.
- **Broaden** ImageJ’s usefulness and relevance across many disciplines of the scientific community.
- **Maintain** backwards compatibility with existing ImageJ functionality.
- **Unify** online resources to a central location for the ImageJ community.
- **Lead** ImageJ development with a clear vision.

It is important to stress that this mission is, and always will be, informed by pragmatism. For instance, much of ImageJ’s existing user community is centered in the biosciences and related life science fields, and the core ImageJ developers and contributors are part of bioimaging laboratories as principal investigators, staff, students, consultants, etc. [3]. As such, ImageJ’s current development directions tend toward addressing problems in bioimaging. However, most image processing algorithms are generally applicable, and there are users of ImageJ in other scientific fields as well. Hence, we wish to avoid pigeonholing the software as a tool for bioimage analysis only, which would implicitly preclude it from being adopted for other purposes. One of our explicit goals is to exploit commonality across scientific disciplines, leaving the door open for others to collaborate and improve ImageJ in cases where doing so is useful.

### Why ImageJ?

Any time a development effort of this scale is undertaken on an existing tool, it is worth evaluating its impact and the decision to invest such resources. The bioimage informatics field [4] is fortunate to have a wide range of software tools available in both commercial and open source arenas [5]. Open-source tools are especially important in science due to their transparency and inherent ability for sharing and extensibility [6]. This need and ability for method sharing has resulted in a plethora of open-source solutions in bioimage informatics, ranging from image acquisition tools such as *μ*Manager [7, 8]; databases such as Bio-Image Semantic Query User Environment (BisQue) [9] and OME Remote Objects (OMERO) [10]; image analysis suites such as Icy [11] and BioImageXD [12]; scientific workflow and pipeline tools such as CellProfiler [13, 14], KoNstanz Information MinEr (KNIME) [15, 16] and Pipeline Pilot [17]; and (3D) rendering applications such as FluoRender [18] and Vaa3D [19]. There are many other open, bioimaging-oriented software packages besides these, including solutions written in powerful scripting platforms such as R, Python and MATLAB. With such an extensive array of tools, does it make sense to invest in an updated ImageJ platform, rather than building on some combination of more recent tools?

The ImageJ2 project aims to do both, by rearchitecting ImageJ as a shared platform for integration and interoperability across many bioimaging software packages. ImageJ has a unique niche in that it is not a monolithic or single-purpose application, but rather a platform for discovery where the bench biologist can adapt and deploy new image analysis methods. Historically, ImageJ 1.x has been popular due to not only pre-designed tools developed for a single purpose and regularly maintained and updated, but also its powerful yet approachable plugin and macro environments that have enabled hundreds of groups to generate results through the development of thousands of customized plugins and scripts [2, 20, 21]. It is this ability for sharing, and the desire to engage the professional and amateur developer alike, that drove the development for ImageJ2. The new version of ImageJ is a platform for extensibility and cross-application cooperation, broadening the scope of ImageJ into a new effort called SciJava [22]: a collaboration of projects striving to cooperate and build on one another both socially and technically. It is our intent that with the developments detailed in this paper, the synergy between these tools, which include ImageJ, KNIME, CellProfiler, OMERO and others, will only increase as each tool continues to evolve along with current avenues of scientific inquiry, benefiting not only existing users, but new users and communities as well. See Table 1 in the “Results and discussion” section for a detailed breakdown of software that has been successfully integrated with ImageJ.

**Table 1:** ImageJ software integrations

Software	Integration project	Supporting technologies
Apache Groovy [37]	SciJava Scripting: Groovy [101]	-
BeanShell [102]	SciJava Scripting: BeanShell [103]	-
Bio-Formats [51]	SCIFIO-Bio-Formats [104]	SCIFIO-OME-XML [105]
Bio7 (R + ImageJ 1.x) [52]	-	Eclipse [106]
CellProfiler [13]	ImageJ Server* [107]	-
ImageJ 1.x [1]	ImageJ Legacy [108]	ImageJ 1.x Patcher [109], Javassist [47]
ITK [39]	ImageJ-ITK [40]	SimpleITK [110]
JavaScript [111]	SciJava Scripting: JavaScript [112]	Nashorn [113], Rhino [114]
Jupyter Notebook [115]	SciJava Jupyter Kernel [116]	BeakerX ^*‡*^ [117]
KNIME [15]	KNIME Image Processing [16]	-
Kotlin [118]	SciJava Scripting: Kotlin [119]	-
Lisp (JVM) [120]	SciJava Scripting: Clojure [121]	Clojure [122]
MATLAB [123]	SciJava Scripting: MATLAB [124]	matlabcontrol [125]
MATLAB	ImageJ-MATLAB [38]	SciJava Scripting: MATLAB
MiToBo ^*§*^ [126]	-	Alida ^*†*^ [127]
OMERO [10]	ImageJ-OMERO [41]	-
OpenCV ^*¶*^ [86]	IJ-OpenCV [128]	JavaCV [129]
Python (CPython or JVM) [130]	imglib2-imglyb [131]	pyJNIus [132], Jython [133], JyNI [134]
Python (CPython)	imagey [135]	imglib2-imglyb
Python (CPython)	SciJava Scripting: CPython [136]	javabridge [137]
Python (JVM)	SciJava Scripting: Jython [138]	Jython, JyNI
R (JVM) [139]	SciJava Scripting: Renjin [140]	Renjin [141]
REST ^∥^ [142]	ImageJ Server* [107]	Dropwizard [143]
Ruby (JVM) [144]	SciJava Scripting: JRuby [145]	Ruby [144]
Scala [146]	SciJava Scripting: Scala [147]	-
TensorFlow [148]	ImageJ-TensorFlow [149]	-

* Provides cross-language interprocess integration with JavaScript, Python and others.

*†* Advanced Library for Integrated Development of data analysis Applications (Alida).

*‡* Beaker Extensions for Jupyter (BeakerX).

*§* Microscopy image analysis ToolBox (MiToBo).

*¶* Open source Computer Vision library (OpenCV).

∥ REpresentational State Transfer (REST)

### Design goals

The central technical design goals of ImageJ2 can be divided into seven key categories: functionality, extensibility, reproducibility, usability, performance, compatibility and community. In this section, we discuss the goals of ImageJ2 from its outset; for how these goals have been met in practice, see the subsequent sections.

#### Functionality

The overriding principle of ImageJ2 is to create ***powerful*** software, capable of meeting the expanding requirements of an ever-more-complex landscape of scientific image processing and analysis for the foreseeable future. As such, ImageJ needs to be more than a desktop application: it must be a ***modular***, multi-layered set of functions with each layer encapsulated and building upon lower layers. In computer science terminology, ImageJ2 strives to have a proper ***separation of concerns*** between data model and display thereof, enabling use within a wide variety of scenarios, such as headless operation—i.e., running remotely on a server, cluster or cloud without a graphical user interface (UI).

At its core, ImageJ2 aims to provide robust support for ***N-dimensional*** image data, to support domains with dimensions beyond time and space. Examples include: multispectral and hyperspectral images, fluorescence lifetime measured in the time or frequency domains, multi-angle data from acquisition modalities such as light sheet fluorescence microscopy, multi-position data from High Content Screens, and experiments using polarized light. In general, the design must be robust enough to express any newly emerging modalities within its infrastructure.

Finally, it is not sufficient to provide a modular framework—ImageJ2 must also provide ***built-in routines*** as default behavior for standard tasks in image processing and analysis. These core plugins must span a wealth of algorithms for image processing and analysis, image visualization, and image file import and export. Such built-in features ensure users have an application they can apply out-of-the-box.

#### Extensibility

According to a survey of ImageJ users, the greatest strength of ImageJ is its ***extensibility*** [23]. From its inception [1], ImageJ 1.x has had a mechanism by which users can develop their own plugins and macros to extend its capabilities. Two decades later, a plethora of such plugins and macros have been shared and published [20]. It is paramount that ImageJ2 maintains this ease of modification and extension by its user community, and furthermore leverages its improved separation of concerns to actually make user extension easier and more powerful; e.g., if image processing plugins are agnostic to user interface, new interfaces can be developed without a loss of functionality.

A related preeminent concern is ***interoperability***. There is no silver bullet in image processing. No matter how powerful ImageJ becomes or how many extensions exist, there will always be powerful and useful alternative tools available. Users benefit most when information can easily be exchanged between such tools. One of ImageJ2’s primary motivations is to enable usage of ImageJ code from other applications and toolkits, and vice versa, and to support open standards for data storage and exchange.

#### Reproducibility

For ImageJ to be truly useful to the scientific community, it must be not only technically feasible to extend, but also socially feasible, without legal obstacles or other restrictions preventing the free exchange of scientific ideas. To that end, ImageJ must be not only open source, but offer full ***reproducibility***, following an ***open development process*** which we believe is an optimal fit for open scientific inquiry [24]. We want to enable the community to not just use ImageJ, but also to build upon it, with all project resources—revision history, project roadmap, community contribution process, etc.—publicly accessible, and development discussions taking place in public, archived communication channels so that interested parties can remain informed of and contribute to the project’s future directions. Such transparency also facilitates sensible, defensible software development processes and fosters responsibility amongst those involved in the ImageJ project. In particular, the project must be well covered by automated tests, to validate that it produces consistent results on reference data sets.

#### Usability

Modular systems composed of many components often have a corresponding increase in conceptual complexity, making them harder to understand and use. To avoid this pitfall, ImageJ2 employs the idea of complexity minimization: seeking ***sensible defaults*** that make simple things easy, but difficult things still possible. The lowest-level software layers should define the program’s full power, while each subsequent layer reduces visible complexity by choosing default parameters suitable for common tasks. The highest levels should provide users with the simplicity of a “big green button,” performing the most commonly desired tasks with ease—the powerful inner machinery remaining unseen, yet accessible when needed.

To bridge the gap between extensibility and usability, there must be a painless process of installing new functionality: a built-in, configurable ***automatic update mechanism*** to manage extensions and keep the software up-to-date. This update mechanism must be scalable and distributed, such that software developers can publish their own extensions on their own websites, without needing to obtain permission from a central authority.

#### Performance

N-dimensional images and the ever-expanding size of datasets increase the computation requirements placed on analysis routines. For ImageJ2 to succeed, it must accomplish its goals without negatively impacting performance ***efficiency*** in time—e.g., Central Processing Unit (CPU) and Graphics Processing Unit (GPU)—or space—e.g., Random-Access Memory (RAM) and disk. Furthermore, to ensure ImageJ2 meets performance needs for a wide variety of use cases, it should offer choices surrounding usage of available resources, as well as sensible defaults for balancing performance in common scenarios.

Another key consideration for performance is ***scalability***: ImageJ must be capable of operating on increasingly huge datasets. In cloud computing, this requirement is often met via elasticity: the ability to transparently provision additional computing resources—i.e., throw more computers at the problem [25]. We are at the dawn of the “Big Data” era of computing, where both computation and storage are scalable resources which can be purchased from remote server farms. Software like ImageJ which hopes to remain effective for serious scientific inquiry into the coming decades must be architected so that its algorithms scale well to increasingly large data processed in parallel across increasingly large numbers of CPU and GPU cores.

#### Compatibility

There are a vast number of existing extensions—plugins, macros, and scripts—for the original ImageJ 1.x application which have proven extremely useful to the user community [20]. ImageJ2 must continue to support these extensions as faithfully as possible, while also providing a clear incremental migration path to take advantage of the new framework.

#### Community

The principal non-technical goal of ImageJ2 is to serve the ImageJ community as it evolves and grows; to that end, several community-oriented technical goals naturally follow. The ImageJ project must provide ***unified online resources*** including a central community-editable website, discussion forum, and online technical resources for managing community extensions of ImageJ. And the ImageJ application itself must work in concert with these resources—e.g., users should be able to report bugs directly to online issue tracking systems when something goes wrong.

## Implementation

Broadly speaking, ImageJ2 components are classified into one of four domains:

- **SciJava.** The most fundamental layers of ImageJ2 are independent from image processing, but rather provide needed functionality common to many applications. On a technical level, the SciJava core components are a set of standard Java libraries for managing extensible applications. Socially, the SciJava initiative is a pledge among cooperating organizations to work together, reuse code, and synergize wherever possible [26].
- **ImgLib2.** To ensure generality of image analysis, ImageJ2 is built on the flexible ImgLib2 container model [27]. Decoupling the elements of image representation, ImgLib2 components enable general image processing algorithms to be reused, regardless of image type, source, or organization.
- **SCientific Image Format Input and Output (SCIFIO).** SCIFIO components define standards for reading, writing, and translating between image formats [28]. These libraries ensure a broad spectrum of image data can be interpreted in a consistent manner across all SciJava applications.
- **ImageJ.** Low-level components establish image metadata and algorithm patterns, built on the SciJava and ImgLib2 layers. High-level components focus on “end user” tools for working with image data, and include user interfaces, user-facing commands, and the top-level ImageJ application [29].

These layers, taken as a whole, form the **ImageJ software stack** [30], the core set of components upon which ImageJ-based applications are built.

Each domain is itself divided into many individual libraries, each of which targets a particular function. This separation of concerns provides a logical organization which allows targeted reuse and extension of any given functionality of interest.

The following sections describe, in order from lowest to highest level, the essential backbone libraries of ImageJ2. Note that this is not an exhaustive list of components, as many components across these domains provide secondary functions—e.g.: script languages, build management, UI elements, or targeted implementations of specific features.

### SciJava common

The ground floor of the ImageJ software stack is the SciJava Common library [31], providing the core framework for creating extensible applications. The heart of SciJava Common is its **application container**, the Context class. Each Context encapsulates runtime application state: available extensions, open images and documents, user settings, etc. The application container paradigm allows multiple independently configured instances of SciJava applications to run concurrently within the same Java Virtual Machine (JVM).

#### Service framework

The application container consists of a collection of **services**, which are initialized dynamically at runtime. These services provide methods which operate on the system in various ways, such as opening data, manipulating images, or displaying user interface elements on screen. Taken as a whole, these service methods constitute the bulk of the Application Programming Interface (API) of ImageJ. Software developers are free to extend the system with new, needed services and/or override any aspect of behavior provided by existing services. This approach is in contrast to the most common naive design of many software projects, which use global “static” state and functions, whose behavior is difficult or impossible to override or enhance in downstream code.

The SciJava Common library itself provides the most fundamental of these services, such as:

- A **plugin service**, which dynamically discovers available plugins using an index generated at compile time by a Java annotation processor. This plugin index is used to bootstrap the application context, since services are themselves a type of plugin.
- An **event service**, which provides a hierarchical publish/subscribe model for event handling.
- A **log service**, for environment-agnostic data logging.
- An **object service**, which keeps a central typed index of available objects.
- A **thread service**, which manages a thread pool and dispatch thread(s) for code execution.
- An **Input/Output (I/O) service**, for reading and writing of data.
- A **preference service**, for saving and restoring user-specific preferences.

In principle, SciJava Common is similar to frameworks such as Spring [32], offering standard software engineering patterns such as dependency injection (DI) [33] and inversion of control (IoC) [34], but tailored to the needs of collaborative scientific projects like ImageJ. For example, SciJava Common provides a generalized I/O mechanism for opening data from any source, but the library itself has no specific knowledge of how to open eXtensible Markup Language (XML) documents, microscopy image formats, or spreadsheets of numerical results—such functionality is provided by downstream components as SciJava plugins (see next section).

#### Plugin framework

SciJava Common provides a unified mechanism for defining **plugins**: extensions which add new features or behavior to the software, and/or modify existing behavior. Plugins are discovered by the system at runtime, and ordered according to assigned priorities and types, forming type hierarchies: structural trees that define how each individual plugin fits into the system. The typical pattern for a desired sort of functionality is to define a dedicated plugin type, then implement plugins that fulfill that operation in various ways. SciJava Common is designed to make virtually any aspect of an application extensible. Some of the most critical plugin categories and types include:

**Core extensibility**

- **Service** – A collection of related functionality, expressed as an API. SciJava services are singletons with respect to each application context. For example, each instance of ImageJ2 has exactly one AnimationService responsible for managing animations, with methods to start and stop animations, select the dimension over which to animate, adjust frame rate, and other options. Note that while the behavior of services can certainly be modified by extensions, doing so is primarily the domain of advanced developers looking to radically alter behavior of the system.
- **IOPlugin** – A plugin that reads data from and/or writes data to a location, such as a file on disk. For example, the SciJava layer provides I/O plugins for common text formats such as Markdown [35], while the SCIFIO layer provides an I/O plugin for image formats.

**Modules**

- **Command** – An operation, more generally known as a SciJava ***module***, with typed inputs and outputs. These modules typically appear in the menu system of the application’s user interface, but can be exposed via interoperability mechanisms in many other ways, such as nodes in KNIME or modules in CellProfiler [13]. When ImageJ users talk about “writing a plugin” they usually mean a Command. See “Module framework” below for more on SciJava modules.
- **ScriptLanguage** – A programming language for SciJava scripts. Each script language plugin provides the logic necessary to execute scripts written in that language (e.g., JavaScript or Python) as SciJava modules with typed inputs and outputs, in a similar way to commands. It also makes it possible to express operations as code snippets that can be reused in scripts to repeat those operations.
- **Converter** – A plugin which transforms data from one type of object to a different type of object. Converters greatly extend the concept of type conversion from what Java provides out of the box to provide automatic conversion in a wide and extensible set of circumstances. For example, it becomes possible for an algorithm to accept a string in place of a floating point numerical value, as long as that string can be parsed to such a value—or to transparently convert between normally-incompatible image data structures from different image processing ecosystems.
- **ModulePreprocessor** – A “meta-module” which prepares modules to run. For example, the LoadInputsPreprocessor populates a module’s inputs with the last-used values as defaults, which can save the user a lot of time. Preprocessor plugins are executed in priority order as part of the module “preprocessing chain” before the module is actually executed.
- **ModulePostprocessor** – A “meta-module” which does something with a module after it has run. For example, the DisplayPostprocessor takes care of displaying the outputs of a module after it has completed execution. Postprocessor plugins are executed in priority order as part of the module “postprocessing chain” after the module is actually executed.

**User interface**

- **UserInterface** – A plugin providing an application UI. These plugins include functionality for creating and showing windows and dialogs. ImageJ2 includes a user interface written in Java’s Swing toolkit which is modeled closely after the ImageJ 1.x design, as well as a UserInterface plugin that wraps ImageJ 1.x itself. But other UIs are equally possible; since a UI is simply a type of plugin, anyone can develop their own SciJava UI without any code changes to the core system. The system is even flexible enough to display multiple UIs simultaneously.
- **Platform** – A plugin which enables customization of behavior based on machine-specific criteria, such as specific flavor of operating system or Java language, including type, architecture, or version. For example, on Macintosh Operating System (macOS), the menu bar appears at the top of the screen, with the About, Preferences, and Quit commands relocated to the Application menu.
- **InputWidget** – A user interface element for harvesting typed inputs. Typically, these widgets are presented as part of a form in a dialog box which prompts the user to fill in input values of a module. In principle, the widgets can be used for anything requiring typed input from the user. For example, a FileWidget allows the user to select a file (java.io.File) on disk, while a ToggleWidget provides a boolean toggle (typically rendered as a checkbox). The SciJava layer provides UI-agnostic interfaces to the common widget types, along with widget implementations corresponding to each supported UserInterface plugin. However, an extension to the system can not only implement its own data structure classes which it uses as inputs to its modules; it can also provide corresponding widgets for those structures, allowing the user to populate them from the user interface in innovative ways.
- **Display** – A plugin for visualizing data. For example, an ImageJ2 ImageDisplay can show two-dimensional planes of N-dimensional image data in a window with sliders for controlling which plane is visible. However, the framework imposes no limits on the sorts of objects that can be visualized; other examples include the TextDisplay, which shows strings, and the TableDisplay, which shows tabular data as a spreadsheet. These plugins are typically used to display a module’s typed outputs (i.e., its results).
- **Tool** – A collection of rules binding user input (e.g., keyboard and mouse events) to display and data manipulation actions. For example, ImageJ2’s PanTool pans a display when the mouse is dragged or arrow key is pressed; the PencilTool draws hard lines on the data within an image display. Many user interfaces render them as icons in the application toolbar.
- **ConsoleArgument** – A plugin that handles arguments passed to the application as command line parameters. This plugin type makes the application’s command line parameter handling extensible—a feature especially important for headless operation sans user interface.

This encapsulation of functionality, coupled with a plugin prioritization mechanic, allows SciJava-based software to be fully customized or extended at any point. An application such as ImageJ is then simply a collection of plugins and services built on top of the SciJava Common framework. For instance, the ImageJ Common [36] component introduces new services specifically for opening and displaying images, specializing the functions defined in the lower-level components. Assigning these specialized functions a higher plugin priority creates a natural, flexible ordering of operations. Given that everything from user interfaces to file formats uses the SciJava plugin mechanism, the path for overriding any behavior is clear and consistent.

Finally, to keep the plugin development process as simple as possible, great care is taken throughout the codebase to adhere to interface-driven design with default method implementations whenever possible. This strategy minimizes the amount of code developers are responsible for writing, lowering the barrier to entry for creating and modifying plugins.

#### Module framework

To successfully interoperate with other scientific software, ImageJ algorithms must be decoupled from the various user interfaces and applications which might want to expose them to end users.

The key concept SciJava employs is that of ***parameterized modules***: executable routines with declared input and output parameters of specific types. These modules can take the form of Command plugins or be expressed as scripts written in any supported scripting language (via available ScriptLanguage plugins; see “Plugin framework” above). For example, a user might write the following parameterized Groovy [37] script:



This script accepts two parameters as input—a name and an age—and outputs a greeting based on the input values. Note the typing: the name can be any string of characters, but the age must be an integer value; the greeting is also a string of characters. Note also that this script makes no assumptions about user interface; it is the responsibility of the framework to: A) prompt the user for the input values in the most appropriate way, B) execute the module code itself, and finally, C) process and/or display the output values in the most appropriate way.

As such, this scheme has great potential for reuse across a wide variety of contexts. For example, when running the above script from the ImageJ user interface, a Swing dialog box will pop up allowing the user to enter the name and age values; and after OK is pressed, the greeting will be displayed in a new window. However, when running the script headless from the command line interface, the input values can be passed as command line arguments and the output values echoed to the standard output stream. See Additional file 1: Figure S1 for an illustration. Since many computational tools have this concept of parameterized modules, SciJava developers need only create some general adapter code to integrate the SciJava module framework with a given tool—and then all SciJava modules become automatically available within that tool’s paradigm. We have already implemented such integration for several other tools in the SciJava ecosystem, including CellProfiler, KNIME [16], and the OMERO image server [10].

SciJava Common has an important mechanism which facilitates the extensible and configurable execution of modules: module pre- and post-processing. Developers can write ModulePreprocessor and ModulePostprocessor plugins to extend what happens when a module is executed (see “Plugin framework” above). Moreover, there are also two plugin types built on this module processing mechanism which make it easy to customize and extend how modules behave:

1. The process of collecting module inputs is known as *input harvesting*. The InputWidget plugin type lets developers create widgets to harvest specific types of inputs from the user. In particular, the SciJava project provides Swing widgets for several data types (Additional file 1: Table S1).
  Some inputs are also automatically populated via ModulePreprocessor code. For example, when a single image parameter is declared, an “active image preprocessor” detects the situation, populating the value with the currently active image. In this way, the user does not have to explicitly select an image upon which to operate in the common case, but the module still has semantic knowledge that an image is one of the routine’s input parameters.
2. The process of dealing with outputs after a module executes is known as *displaying*. The Display plugin type lets developers visualize specific types of outputs in appropriate ways. The SciJava layer provides a basic display plugin for text outputs, which shows the text in a dedicated window, while the ImageJ layer provides additional similar display plugins for image and tabular data.

One final SciJava subsystem of note is the *conversion framework*, which provides a general way of transforming data from one type to another. The Converter plugin type lets developers extend SciJava’s conversion capabilities, allowing objects of one type to be used as module inputs of a different type, in cases where the two types are conceptually analogous. For example, data stored in memory as a MATrix LABoratory (MATLAB) matrix can be expressed as an ImageJ image object, even though MATLAB matrices are not natively ImageJ images [38]. When a suitable converter plugin is present, modules capable of operating only on MATLAB matrices become transparently capable of accepting ImageJ images as inputs, thanks to the framework’s auto-conversion. Similarly, a converter between ImageJ and the Insight ToolKit (ITK) [39] images greatly streamlines use of ITK-based algorithms within ImageJ [40].

### ImageJ common

Meeting the needs of contemporary scientific image analysis requires a flexible and extensible data model, including support for arbitrary dimensions, data types and image sizes. To this end, we have chosen to model ImageJ2 images using the ImgLib2 library, which itself provides an extensible, interface-driven design that supports numeric (8-bit unsigned integer, 32-bit floating point, etc.) and non-numeric data types. It also provides great flexibility regarding the source and structure of data. Out of the box, ImgLib2 provides several data sources and sample organizations, including use of a single primitive array (“array image”), one array per plane (“planar image”), and block-based “cell image.” However, the library remains general enough that alternative structures are also feasible. To quote the ImgLib2 article [27]:

The core paradigm [of ImgLib2] is a clean separation of pixel algebra (how sample values are manipulated), data access (how sample coordinates are traversed), and data representation (how the samples are stored, laid out in memory, or paged to disc). ImgLib2 relies on virtual access to both sample values and coordinates, facilitating parallelizability and extensibility.

ImageJ Common provides a unification of the type and storage-independence of ImgLib2 with the SciJava Common plugin framework (described above). A Dataset interface provides the fundamental representation of ImageJ images, collections of images, and corresponding metadata: (ROIs), visualization settings, sample coordinates and physical calibrations, and much more. Also provided are plugins and services for working with these Dataset objects. Together, these classes form the access points for higher-level components to open, save, generate and process these images.

Note that as of this writing, elements of the ImageJ Common data model and corresponding services are still stabilizing. As such, we do not describe these structures in technical detail here.

### SCIFIO

An essential goal of ImageJ2 is to establish universal image analysis routines, with no limits on application; however, the proliferation of proprietary image formats from scientific instruments creates a major obstacle to this ambition. To overcome this issue, the SCIFIO core library establishes a common framework for reading, writing and translating image data to and from the ImageJ Common data model, as well as between domain-specific standard metadata models. SCIFIO builds on the services provided in SciJava Common and ImageJ Common, defining image Format and metadata Translator plugin types to encapsulate the operations necessary to take an image source and standardize it as an ImageJ Dataset.

SCIFIO builds upon SciJava Common’s core I/O infrastructure, which allows it to operate on most data locations independent of their nature. SciJava Common provides a Location interface which acts as a data descriptor, similar to a Uniform Resource Identifier (URI). This Location interface is specialized according to the nature of the data; for example, a URLLocation identifies data served by a remote Uniform Resource Locator (URL), while an OMEROLocation (part of the ImageJ-OMERO integration [41]) identifies an image from an OMERO server. For data locations whose raw bytes can be accessed randomly and/or sequentially (e.g., remote URLs, but not OMERO images), SciJava Common provides a DataHandle plugin type which enables such access. The core library provides DataHandle plugins for several kinds of data locations, including files on disk, remote URLs, and arrays of bytes in local computer memory. Developers can easily create new DataHandle plugins which provide random access into additional sorts of locations, and SCIFIO will be able to use them transparently without any changes to existing Format or Translator plugins.

The Format plugin API is architected to support reading and writing of image data in chunks, which provides scalability. It is no longer necessary to have a large quantity of computer RAM to work with large images—SCIFIO reads the data from the source location on demand, paging it into and out of memory as needed. SCIFIO’s caching mechanism persists any changes made to image pixels, even when chunks leave memory, by using temporary storage on disk.

SCIFIO Translator plugins provide the means to translate not only between image formats, but between common metadata models of various scientific disciplines. For example, the Open Microscopy Environment (OME) defines a data model called OME-XML [42], for which the SCIFIO-OME-XML component provides a suite of translators to and from ImageJ Common data structures. In this way, SCIFIO has the potential to bridge interoperability gaps across various discipline-specific scientific software packages.

Further details about SCIFIO can be found in the BioMed Central (BMC) Bioinformatics software article “SCIFIO: an extensible framework to support scientific image formats” [28].

### ImageJ ops

**Fig. 1:**
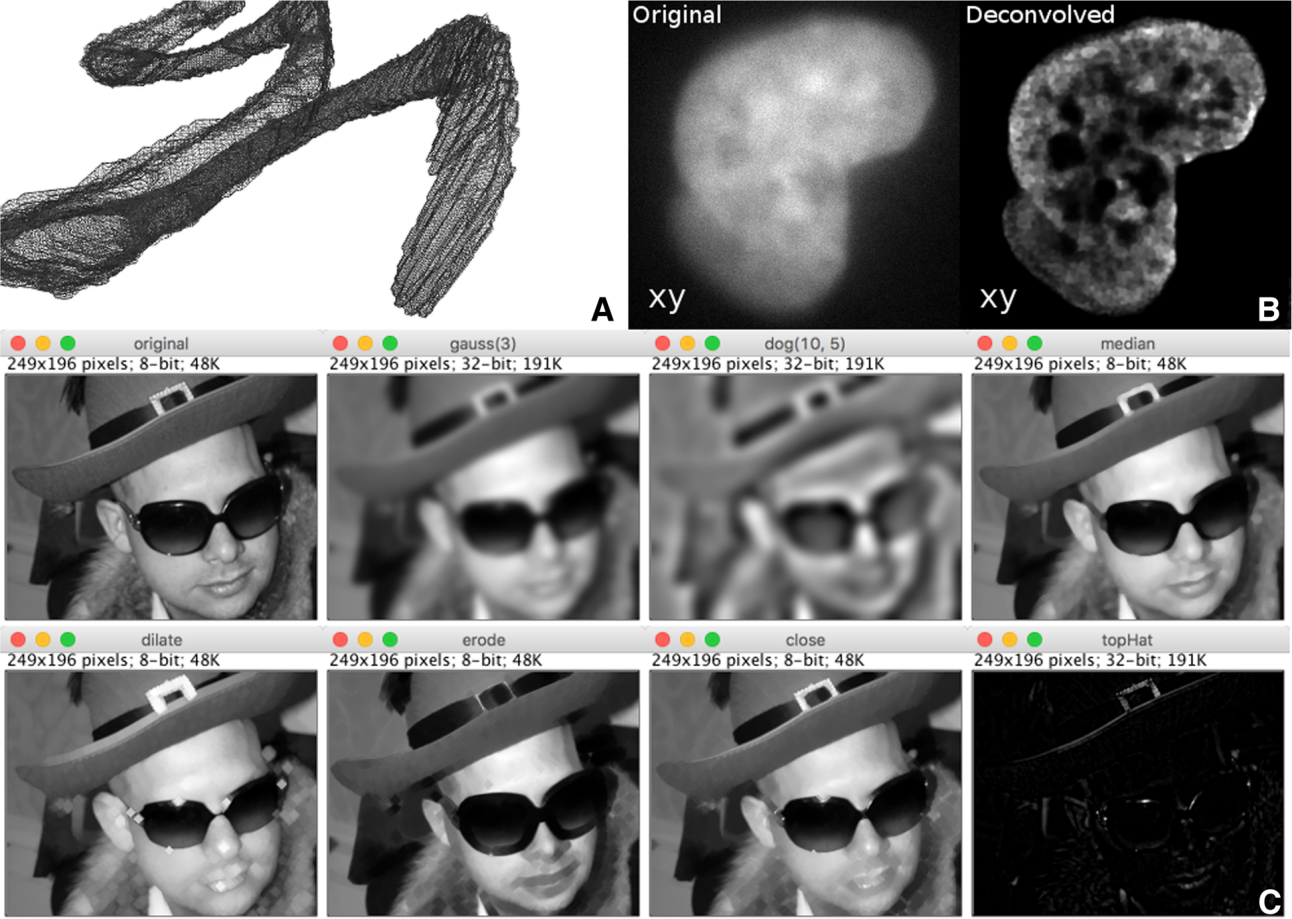
Examples of image processing algorithms available in ImageJ Ops. Panel **a** (top left): 3D wireframe mesh of ImageJ’s Bat Cochlea Volume sample dataset [94], computed by the geom.marchingCubes op, an implementation of the marching cubes algorithm [95], visualized using MeshLab [96]. Credit to Kyle Harrington for the figure, Tim-Oliver Buchholz for authoring the op, and Art Keating for the dataset. Panel **b** (top right): Richardson-Lucy Total Variation deconvolution [97] of the Stellaris FISH dataset #1 [98], computed by the deconvolve.richardsonLucyTV op. Credit to Brian Northan for authoring the op and figure [99], and George McNamara for the dataset. Panel **c** (bottom): Grayscale morphology and neighborhood filter operations on Fiji’s New Lenna sample image, using a diamond-shaped structuring element with radius 3. Credit to Jean-Yves Tinevez, Jonathan Hale and Leon Yang for authoring the ops

ImageJ’s ultimate purpose is image processing and analysis. To that end, we have crafted the ImageJ Ops component: ImageJ2’s shared, extensible library of reusable image processing operations. As of version 0.33.0, the core Ops library provides 788 Op plugins across nearly 350 types of ops in more than 20 namespaces, covering functionality such as: image arithmetic, trigonometry, Fourier transformations, deconvolution, global and local thresholding, image statistics, image filtering, binary morphological operations, type conversion, image transformations (scaling, rotation, etc.)—even 2- and 3-dimensional geometric operations such as marching cubes 3D mesh generation (see Fig. 1 for examples). A thorough treatment of available ops can be found in the ImageJ Ops tutorial notebook [43].

ImageJ Ops was conceived with three major design goals: 1) easy to use and extend; 2) powerful and general; and 3) high performance. To achieve all three of these goals, Ops utilizes a plugin-based design enabling “extensible case logic.” Ops defines a new plugin type, Op, each of which has a name and a list of typed parameters, analogous to a function definition in most programming languages. When invoking an op, callers typically do not specify the exact Op plugin to use, but instead specify the operation’s name and arguments; the Ops framework then performs a *matching* process, finding the optimal fit for the given request. For example, calling math.add with a planar image and a 64-bit floating point number leads to a match of net.imagej.ops.math.ConstantToPlanar Image.AddDouble, which adds a constant value to each element of an image, whereas calling math.add with two planar images results in a match of net.imagej.ops.math.IIToIIOutputII.Add, which adds two images element-wise.

This scheme is similar to—but more powerful than—the method overloading capabilities of many programming languages: op behavior can be further specialized by tailoring Op implementations for specific subclasses, generic parameters, and Converter substitutions (see “SciJava common” above). Consider an op sqrt(image), which computes the element-wise square root of an image. If we implement this op as sqrt(Dataset), we miss out on performance optimizations for ArrayImg, an ImgLib2 container type where the entire collection of image samples is stored in a single Java primitive array. However, if we only implement sqrt(ArrayImg), we are restricted in supported data types, since not all images can be stored in such a manner. The power of the Ops matching approach is that both of these and more can coexist simultaneously and extensibly, and the most specific will always be selected at runtime.

Furthermore, as algorithm implementations increasingly become available for the GPU via libraries such as Open Computing Language (OpenCL) [44] and Nvidia’s Compute Unified Device Architecture (CUDA) [45], as well as for clusters via libraries such as Apache Spark [46], such implementations could also be expressed as ops so that they can be selected automatically based on the currently available hardware environment.

The Op plugin type extends SciJava’s Command, and therefore all ops are SciJava parameterized modules, usable anywhere SciJava modules are supported—see the “Module framework” section in “SciJava common” above. Like standard modules, an op declares typed inputs and outputs. However, unlike modules in general, an op must be a “pure function” with a fixed number of parameters and no side effects; i.e., it must be deterministic in its behavior, operating only on the given inputs, and populating or mutating only the given outputs. These restrictions provide some very useful guarantees which allow the system to reason about an op’s use and behavior; e.g., after computing an op with particular arguments once, the result can be cached to dramatically improve subsequent time performance at the potential expense of additional space. Properly constructed ops will also always be usable headless because they do not rely on the existence of UI elements.

**Op chaining and special ops** It is often the case in image processing that an algorithm can be expressed as a composition of lower level algorithms. For example, a simple difference of Gaussians (“DoG”) operation is merely two Gaussian blur operations along with a subtraction:

*dog*(*image*,*σ*_1_,*σ*_2_)=*sub*(*gauss*(*image*,*σ*_1_),*gauss*(*image*,*σ*_2_))

For users calling into the Ops framework via scripting, the core library provides an eval op backed by SciJava’s expression parser library, which enables executing such sequences of ops via standard mathematical expressions, including use of unary and infix binary operators.

For developers, the Ops library provides a mechanism for efficient *chaining* of ops calls. An op may declare other ops as inputs, resulting in a tree of ops which are resolved when an op is matched; the matched op instance can then be reused across any number of input values. In this way, very general operations can be created to address a broad range of use cases—e.g., the map operation provides a unified way of executing an op such as math.sqrt(number) element-wise on a collection (e.g., an image) whose elements are numbers. Indeed, in the case of DoG, the Ops library’s baseline implementation takes an image as input, along with two filter.gauss ops and a math.sub op, and then invokes them on the input image. The baseline stats.mean implementation is similar, built on the stats.sum, stats.size and math.div ops. Higher level DoG ops provide sensible defaults, enabling calls like dog(image, sigma1, sigma2) to work, making common operations simple, while leaving the door open for additional customization as needed.

To facilitate type-safe and efficient chaining of ops, the Ops library has a subsystem known as *special ops*. Such special ops are specifically intended to be called repeatedly from other ops, without needing to invoke the op matching algorithm every time. This repeat usage is achieved in a type-safe and efficient way by explicitly declaring the types of the op’s primary inputs—i.e., the inputs whose values can be efficiently varied across invocations of the op—as well as the type of the op’s primary output.

Special ops have two major characteristics beyond regular ops. First, each special op has a declared *arity*, indicating the number of primary inputs, which are explicitly typed via Java generics and can thus efficiently vary across invocations of the op. Three arities are currently implemented: *nullary* for no inputs, *unary* for one input, and *binary* for two inputs. It is important to note that unlike a formal mathematical function, a unary special op may have more than one input parameter—the “unary” in this case refers to the number of explicitly typed parameters intended to vary when calling an instance of the op multiple times. For instance, in the DoG example above, the baseline DoG is declared as a unary op, so that the input image can vary efficiently while the sigmas etc. are held constant in value.

Secondly, every special op is one of three kinds:

- A *function* operates on inputs, producing outputs, in a way consistent with the functional programming paradigm. Inputs are immutable, and outputs are generated during computation and subsequently also immutable. Functions are very useful for parallel processing since they are fully thread-safe even when object references overlap—but this safety comes at the expense of space, since they offer no way to reuse preallocated output buffers.
- A *computer* is similar to a function, but populates a preallocated output object instead of generating a new object every time. Computers have many of the same advantages of functions, but provide the ability to reuse preallocated output buffers to improve efficiency in space and time.
- An *inplace* op mutates its input(s) in place—i.e., its input and output are the same object. Inplace ops are highly space efficient, but lack the mathematical guarantees of functions and computers, since they destroy the original input data.

Some ops are implemented as *hybrids*, offering a choice between two or more of the function, computer and inplace computation mechanisms. Users of the ops library—even advanced users—will rarely if ever need to know about this implementation detail, but for developers crafting new ops, it is convenient to have unifying interfaces which provide common logic for combining these paradigms. See Additional file 1: Table S2 for a complete breakdown of the special op kinds and arities.

### ImageJ legacy

To maximize backwards compatibility with ImageJ 1.x, ImageJ2 must continue to provide access to the complete existing UI and API with which ImageJ users are familiar, while also making all new ImageJ2 features available for exploration and use. Furthermore, to bridge the gap, ImageJ2 must provide improved functionality transparently when possible, as well as support seamless “mixing and matching” of the two respective APIs. In this way, ImageJ2 can enable gradual migration to the more powerful capabilities of ImageJ2, while empowering developers’ contributions to the framework to be immediately effective. To achieve this goal, we identified the major functional pathways of ImageJ 1.x and reworked them to delegate first to ImageJ2 equivalents, falling back on the old behavior if needed.

There are two ImageJ components dedicated to maintaining backwards compatibility with ImageJ 1.x. The lower level of the two is the IJ1-patcher: using a tool called Javassist [47] to perform an advanced Java technique known as bytecode manipulation, ImageJ 1.x code is modified at runtime to expose callback hooks at critical locations—e.g.: when opening images with *File* *⊳* *Open…*, closing the ImageJ application, or displaying UI components. These hooks are built using the SciJava plugin infrastructure, allowing new behavior to be injected into ImageJ 1.x despite the fact that it was not designed to support such extensibility. In essence, ImageJ2 “rewrites” portions of ImageJ 1.x at runtime to make integration possible. This approach is necessary because altering ImageJ 1.x directly to enable such hooks would break backwards compatibility with existing macros and plugins, ruining established scientific workflows which have otherwise remained functional across many years.

By default, these hooks are exploited to inject ImageJ2 functionality in the second compatibility layer: ImageJ Legacy. ImageJ2 intercepts an ImageJ 1.x request and attempts to delegate to its own routines. For example, in our implementation of the *File* *⊳* *Open…* hook, we use the SciJava I/O service, which provides extensible support for data types via SciJava I/O plugins. This allows the full power of SCIFIO to be called automatically by *File* *⊳* *Open…* without requiring users to select individual loader plugins. In this way, ImageJ2 exposes new “seams” which provide extensibility points not available in the standalone ImageJ 1.x project [48].

A second major function of ImageJ Legacy is to provide a wrapping legacy UI: an ImageJ2 UserInterface plugin that reuses the ImageJ 1.x UI, but maintains synchronization between respective data structures. For example, consider the ImagePlus structure in ImageJ 1.x and its equivalent, the Dataset, in ImageJ2. By default, an ImagePlus and Dataset could not be interchanged; they have different Java class hierarchies, and with ImageJ2’s expanded data model, a Dataset is more expressive than an ImagePlus. However, requiring plugins to “select one” would impose a technical barrier, even if both structures are available in the same application. Thus, the legacy UI notes when either an ImagePlus or a Dataset is created and ensures a complementary instance is mapped, via SciJava Converter plugins. This brings the ImageJ 1.x and ImageJ2 worlds closer together: when an image is opened, it can be used by plugins that would take an ImagePlus or a Dataset regardless of whether that image was opened via an ImageJ 1.x or ImageJ2 mechanism. Furthermore, because conversion is handled in the ImageJ Legacy layer, individual plugins do not require knowledge of the synchronization.

Shared image data structures are but one aspect of the legacy UI’s synchronization. Others include logging, notification, and status events—essentially all UI events are mapped across paradigms. Whenever possible, these conversions are achieved using an adapter class that implements a common interface (e.g., Dataset), which wraps the object of interest (e.g., ImagePlus) by reference. This approach enables information to be translated between ImageJ 1.x and ImageJ2 structures on demand, while minimizing the performance impact. Wrapping by reference also mitigates the burden of updates; once synchronization is established, changes to the underlying object are automatically reflected in the wrapper.

### ImageJ updater

**Fig. 2:**
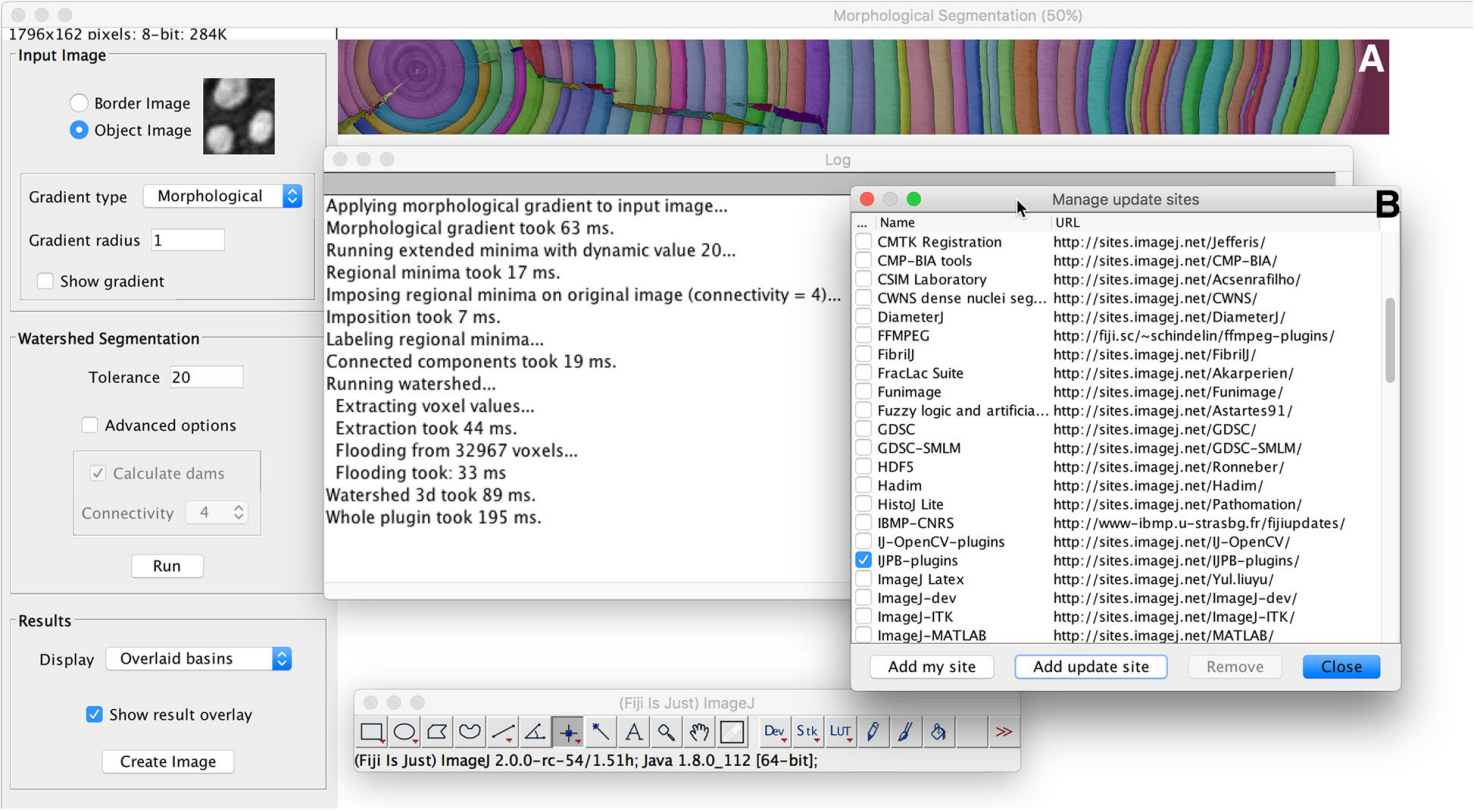
ImageJ update sites provide additional functionality to ImageJ. The Morphological Segmentation plugin, part of the MorphoLibJ plugin collection [100], easily segments the rings of ImageJ’s Tree Rings sample dataset (panel **a**). The MorphoLibJ plugins are installed into the Fiji distribution of ImageJ by enabling the IJPB-plugins update site (panel **b**). Credit to David Legland and Ignacio Arganda-Carreras for authoring the plugins

The ImageJ Updater is the mechanism by which the available and installed components of ImageJ are managed. At its core, the Updater is a flexible component for tracking ImageJ update sites: endpoints containing versioned collections of files. Users can pick and choose which update sites they wish to enable, with ImageJ’s core functionality offered on the base “ImageJ” update site, which is on by default. Distributions of ImageJ such as Fiji Is Just ImageJ (Fiji) [49] extend this base with additional functionality (Fig. 2) in the form of more plugins, scripts, sample images, color lookup tables (LUTs), etc., leveraging the ability to override ImageJ’s base behavior using SciJava’s plugin priority mechanism (see “SciJava common” above for details).

The Updater stores metadata in a db.xml.gz file in the root of each update site, which describes the files that are part of that update site, including checksums and timestamps for all previous versions. In this way, the Updater can tell whether each local file is: A) an up-to-date tracked file; B) an old version of a tracked file; C) a locally modified version of a tracked file; or D) an untracked file. Update sites are served to users over Hypertext Transfer Protocol (HTTP). Developers may upload files to an update site via an extensible set of protocols, as defined by Uploader plugins. The core ImageJ distribution includes plugins for Secure SHell (SSH), Secure CoPy (SCP), Secure File Transfer Protocol (SFTP) and Web Distributed Authoring and Versioning (WebDAV), but in principle, the Updater makes no assumptions about how files are uploaded.

The db.xml.gz structure was originally designed for use with the Fiji Updater, the ImageJ Updater’s predecessor. The logic of the Fiji Updater was migrated into the core of ImageJ2, with backwards compatibility preserved for existing Fiji installations. As part of that migration, the Updater was heavily refactored to be UI agnostic, such that additional user interface plugins for the Updater could be created which leverage the same core. Out of the box, ImageJ provides two different user interfaces for the Updater: a command-line tool intended for power users and developers, and a Swing UI intended to make updating easy for end users. When ImageJ is first launched, it automatically runs the “Up-to-date check” command, which then displays the Updater UI if updates are available from any of the currently enabled update sites.

## Results and discussion

ImageJ has transformed from a single-user, single-bench application to a versatile framework of extensible, reusable operations. In the following sections, we discuss how each core aspect of ImageJ2 has impacted community usage and how we expect these qualities to shape future developments.

### Functionality

The architecture of ImageJ2 enables it to meet current and future demands in image analysis.

**Dimensions.** Using ImgLib2 opens up caching options for operating on extremely large images, an area in which ImageJ 1.x has previously struggled. ImageJ 1.x is inherently limited to five dimensions (X, Y, Z, time, and channel) with fewer than 2^31^ pixels per XY plane, e.g. a 50,000×50,000 plane being too large to represent. ImageJ 1.x allows composite images, but is constrained to a maximum of seven composited channels. ImageJ2’s N-dimensional data model supports up to 2^31^−1 dimensions, each with up to 2^63^−1 elements, and composite rendering over any dimension of interest regardless of length. There are several preset dimensional axis types, and new types can also be defined as needed. When visualizing multi-channel data, each channel can now have its own LUT without constraint.

**Fig. 3:**
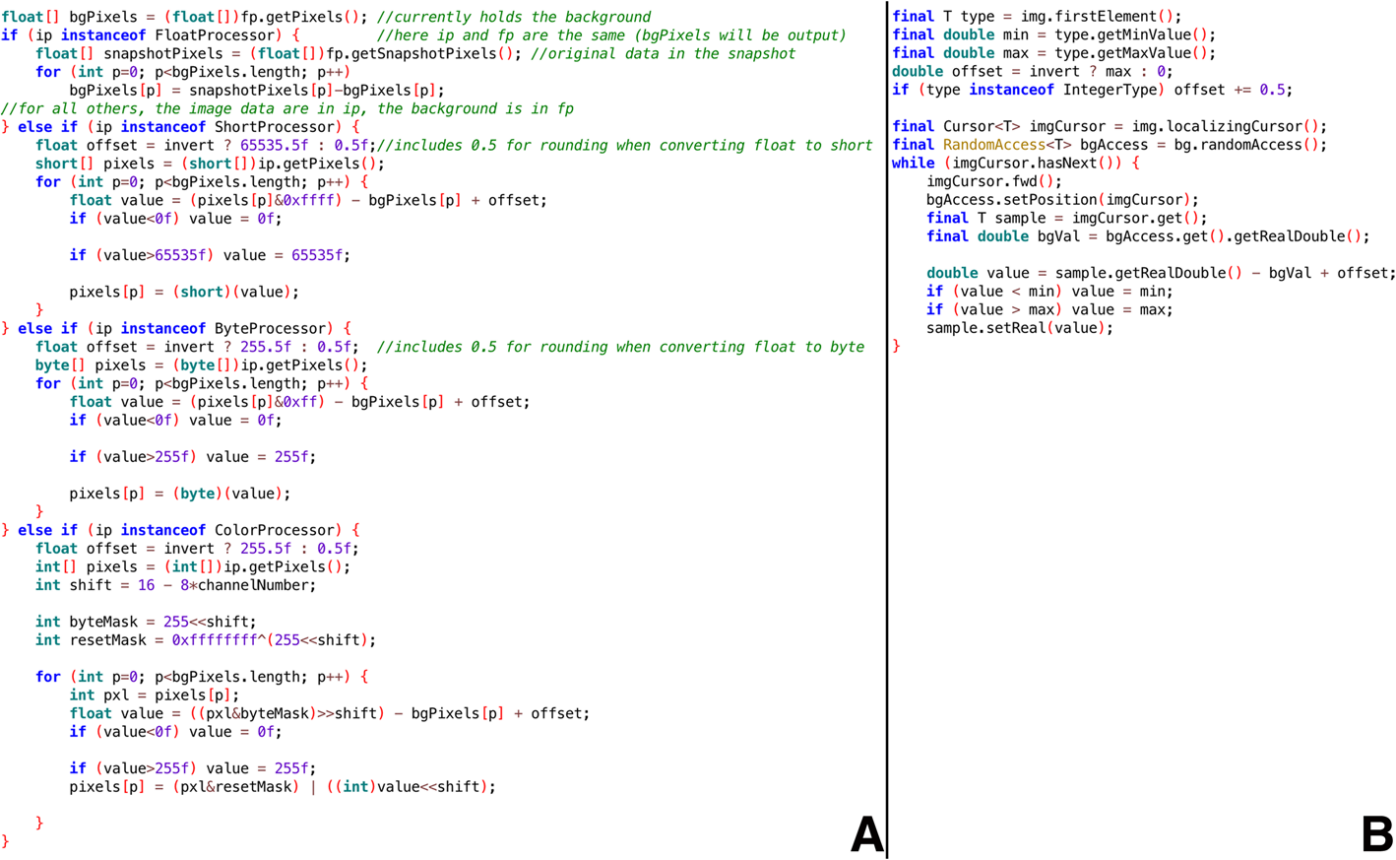
ImageJ 1.x case logic compared to a unified ImgLib2 implementation. Panel **a** (left) shows the ImageJ 1.x implementation of a rolling ball background subtraction method, part of the ij.plugin.filter.BackgroundSubtracter class. Panel **b** (right) shows an equivalent implementation using ImgLib2, without the need for extensive case logic

**Types.** ImageJ 1.x supports only five image types for representing sample values: 8-bit unsigned integer grayscale, 8-bit with a color lookup table, 16-bit unsigned integer grayscale, 32-bit floating point, and a 32-bit integer-packed color type representing three 8-bit unsigned color channels: red, green, and blue. Furthermore, this support is highly static, sometimes requiring case logic for algorithms to properly handle each of desired image types independently. In contrast, ImgLib2 is explicitly designed to facilitate algorithms developed agnostic of image type (Fig. 3). ImageJ2 already supports over twenty different image types (Additional file 1: Table S3), including arbitrary precision integer and decimal types, and further types are definable using SciJava Common’s flexible plugin framework. SciJava Converter plugins also extend the reach of ImageJ2-based algorithms even further into additional data structures, such as MATLAB matrices [38] and ITK images [40].

**Storage.** The prime example of an alternate storage source in ImageJ 1.x is the virtual stack, allowing image slices to be read on demand—e.g., if the image would not normally fit in memory. However, ImageJ 1.x commands must explicitly account for whether or not they can operate on a virtual stack, requiring a proliferation of case logic and complexity. ImageJ2 takes advantage of ImgLib2’s extensible container system, which enables data to be stored flexibly: as files on disk, remote URLs, within a database, generated on-the-fly, etc. Such routines can even be used with pixel and storage types implemented well after their creation without having to change the original implementation. As image acquisition sizes increase, we expect virtualized image data to be particularly critical to the future of image analysis. The SCIFIO library already provides an ImgLib2 image type (“SCIFIO cell image”) that supports block-based read/write caching from disk, effectively behaving as a writeable virtual stack.

**Regions of interest (ROIs).** Like ImageJ 1.x, ImageJ2 provides support for *ROIs*, which are functions that identify samples upon which to operate, as well as *overlays*, which are visuals (e.g., text) superimposed for visualization. ImageJ2 builds upon the ROI interfaces of ImgLib2, allowing for any number of simultaneous ROIs and overlays to be associated with a particular image without the need for additional tools like ImageJ 1.x’s global ROI Manager window.

Because ROIs are part of the core ImgLib2 library, it is possible to process subsets of images identified by one or more ROIs using an ImgLib2-based algorithm, and the Ops library can process data within a ROI as a single functional operation. This continues ImageJ2’s migration towards image processing algorithms that need not add explicit case logic—e.g., to handle ROIs separately—but instead simply provide a pixelwise function, or iterate using ImgLib2’s generic iteration mechanism. In this way, we continue to reduce the effort and complexity of ImageJ2 plugins, while increasing their utility and application.

**Modularity.** ImageJ 1.x was developed with a “single computer, single user, single operation” in mind. Although ImageJ 1.x can be used as a library, it will always be a single unit that cannot be decoupled from its dependencies, which are implicit in its source code. ImageJ2 has succeeded in building a cohesive application from encapsulated, modular components unified by the SciJava plugin framework. Each component is independently deployed and accessible via the build automation tool Maven [50], allowing developers to pick and choose the individual pieces relevant to them—be it the ImageJ application, a particular scripting language, image format, or the SciJava Common core. SciJava-based projects inherit a “bill of materials” which enables components to be combined at versions known to be compatible with each other [30]. We have already seen the benefits of this modularity—for example, the use of the SCIFIO library in KNIME Image Processing (KNIP) to produce images compatible with ImageJ2 commands.

**Ops.** The ImageJ Ops library is the centerpiece of ImageJ2, bringing Java’s mantra of “write once, run anywhere” to image processing algorithms. Ops provides a wealth of image processing algorithms to users, accessible in a unified way that empowers developers to transparently extend and enhance the behavior and capabilities of each operation. It is critical to appreciate that each type of op (more than 350 different operations as of version 0.33.0) represents a potential extension point for optimizing existing parameters, or supporting new ones. In contrast to algorithms coded using ImageJ 1.x data structures, all ops work without modification on all image types (Additional file 1: Table S3) and containers, including those not yet in existence. As the Ops project is a very active collaboration across several institutions including the University of Konstanz, University of Wisconsin-Madison and others, we expect the core library to continue to grow in both available functionality and use within the community.

### Extensibility

**Plugins.** The ImageJ2 plugin framework, built on top of SciJava Common, is a modular and extensible infrastructure for adding features. Plugins can now take many forms, including image processing operations, new tools, and even completely new displays. In ImageJ 1.x there are three kinds of plugins: 1) the standard PlugIn, which provides a freeform run(String arg) method; 2) PlugInFilter, which processes images one plane at a time; and 3) PlugInTool, which adds a function to the toolbar. In ImageJ2, these ideas are expressed in the form of Command, Op and Tool plugins, respectively—although these plugin types have many advantages over their ImageJ 1.x analogues: type-safe chaining of operations, dynamic selection of ops based on arguments, UI agnosticism, etc. Furthermore, many other types of plugins are available as well, and the flexibility of the SciJava plugin framework also allows for additional new types of plugins to be defined.

**Modules.** The ImageJ application’s menu structure is made up of SciJava modules—most commonly commands and scripts. Thus, scripts and Command plugins are probably the most common points of extension for developers exploring the ImageJ2 architecture. Writing such extensions in ImageJ2 is much simpler than in ImageJ 1.x, which requires each extension to explicitly create its own dialog box to collect user input. In ImageJ2, the use of parameters results in more declarative extensions, freeing software developers from the need to explicitly ask the user for input values in the vast majority of cases, and substantially reducing boilerplate and UI-specific code, making commands shorter and easier to understand (see Fig. 5 in “Usability” below). Moreover, this mechanism makes ImageJ2 modules truly independent of the user interface, allowing them to work with any UI or headlessly. The module simply declares its inputs and outputs using the appropriate parameter syntax, and lets ImageJ do the rest.

**Formats.** In an open source image analysis program like ImageJ, an extensive collection of supported image formats is necessary to maximize relevance and impact across the community. ImageJ 1.x provided a central HandleExtraFileTypes class to enable extensibility, but required direct modification of this class to do so, resulting in many third parties each shipping their own modified version. Only one modification could “win,” effectively breaking any other supported formats. To fill this role in ImageJ2, the SCIFIO library provides extensible image format support tailored to the ImageJ Common data model.

As of version 0.29.0, the core SCIFIO library provides a collection of more than 30 open formats, and also includes a wrapping of the Bio-Formats library [51], which enables a wide variety of supported images throughout all ImageJ operations. Furthermore, SCIFIO enables developers to create their own Format plugins to smoothly integrate support for new proprietary formats and metadata standards without modification of core functions or proliferation of one-off format commands.

**Image processing.** ImageJ’s main purpose is effective and extensible image processing; therefore, ImageJ’s extension mechanism for image processing algorithms must be one of its central features. ImageJ2’s op matching subsystem offers extensible case logic: an Op plugin can be written to add a new algorithm, to extend an existing algorithm to support new data structures, or to make an algorithm more efficient for specific data types, all without impacting previously written code. As the Ops library matures, we expect to see new Op implementations along all of these lines in existing third-party suites, conveniently shipped to users via ImageJ update sites. Hence, unlike in ImageJ 1.x, existing user scripts using the Ops library will automatically benefit from new performance-enhancing ops.

**User interface.** ImageJ 1.x’s user interface is written in Java Abstract Windowing Toolkit (AWT) with many assumptions throughout the codebase relying on this fact. Hence, ImageJ 1.x is only limitedly usable in a headless way (e.g., for image processing on a server cluster). Normally, ImageJ 1.x cannot be used headless at all: some lynchpin ImageJ 1.x classes—notably ij.ImageJ and ij.gui.GenericDialog—derive from java.awt.Window, and such classes cannot be instantiated when running in headless mode. Fortunately, the ImageJ Legacy layer’s runtime patcher rewrites affected ImageJ 1.x classes to derive from non-AWT window classes when in headless mode; as such, ImageJ2 makes headless execution of ImageJ 1.x scripts feasible.

Furthermore, ImageJ 1.x’s reliance on AWT also limits its ability to be embedded into other applications using different UI frameworks, such as Swing or Eclipse Standard Widget Toolkit (SWT). While some applications have succeeded in doing so [52], the amount of work required in response to each ImageJ 1.x update is considerable, since many changes must be made to the ImageJ 1.x core source code.

In contrast, ImageJ2’s separation between the underlying data model and the user interface enables it to run headless or within a variety of different user interface paradigms with no changes to the core. Developers can create their own plugins to provide alternative user interfaces. ImageJ2 is even capable of displaying multiple UIs simultaneously in the same Java runtime. Adding support for a new UI now only requires writing a new UserInterface plugin and corresponding display and widget plugins. As one of the most common questions about ImageJ from software developers is how to customize the ImageJ UI, we believe that this improved user interface framework will yield substantial future dividends.

**Fig. 4:**
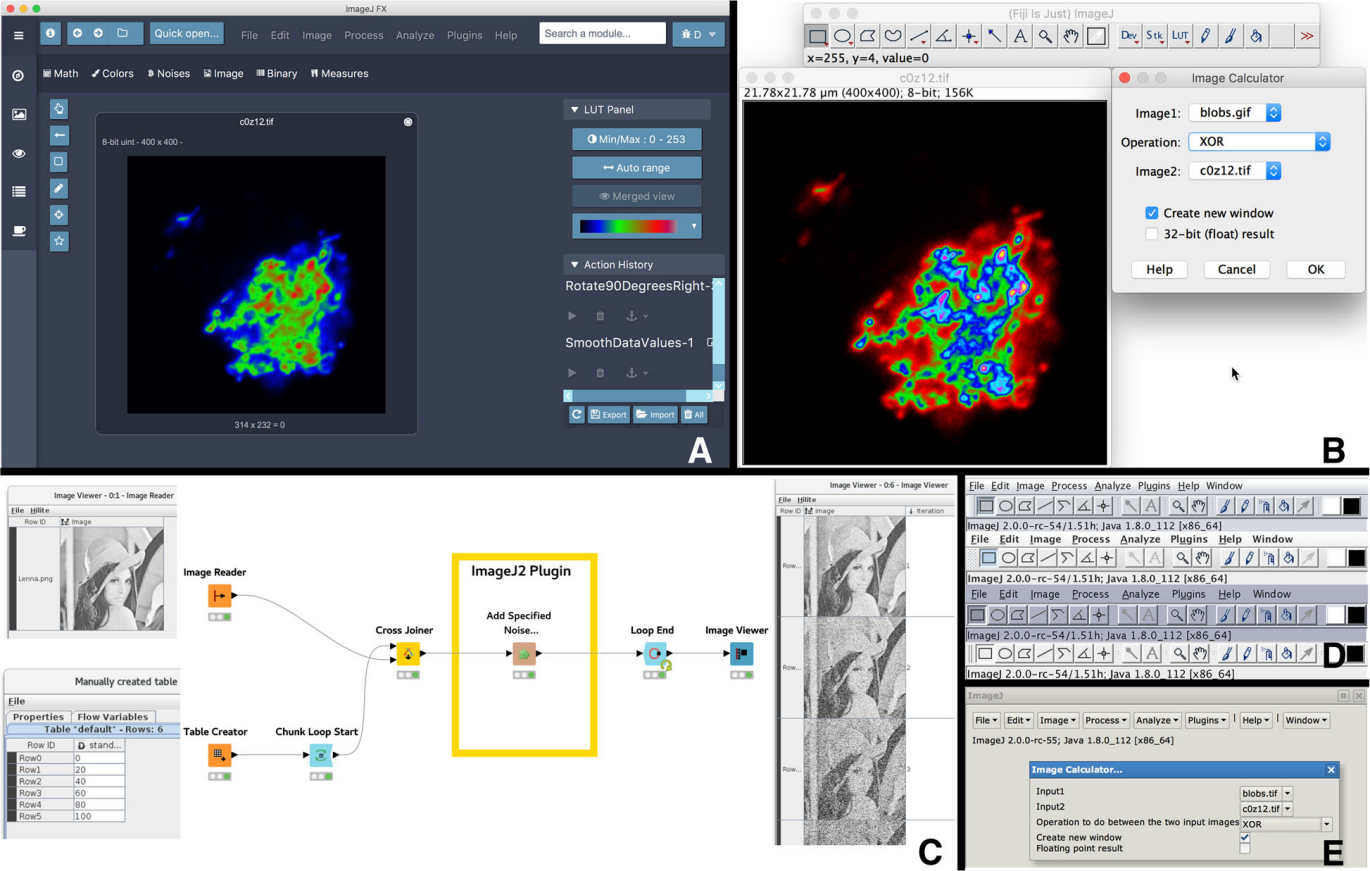
Side-by-side comparison of ImageJ2-based user interfaces and integrations. Panel **a** (top left): ImageJFX, a JavaFX-based user interface built on ImageJ2. Panel **b** (top right): ImageJ2’s default user interface, the ImageJ Legacy UI, which wraps ImageJ 1.x. Panel **c** (bottom left): Example KNIME workflow utilizing ImageJ2 image processing nodes. Panel **d** (middle right): Swing UI prototype, closely modeled after ImageJ 1.x so that it remains familiar to existing users, in various Java “Look & Feel” modes. Panel **e** (bottom right): A proof-of-concept Apache Pivot user interface. The ImageJFX and ImageJ Legacy UIs display an XY slice of ImageJ’s Confocal Series sample dataset (dataset courtesy of Joel Sheffield), which has been rotated, smoothed and colorized

While the current flagship user interface for ImageJ2 is still the ImageJ 1.x UI via the ImageJ Legacy component, ImageJ2 also has a Swing user interface modeled after the ImageJ 1.x UI, but which stands alone with no dependence on ImageJ 1.x code. We have been successful in “reskinning” this Swing UI with various Java Look & Feels (L&Fs), including the Metal, Motif, Nimbus, Aqua, Windows and GIMP ToolKit (GTK) L&Fs. Furthermore, we explored proof-of-concept UI implementations in other frameworks, such as Eclipse’s SWT, Java AWT sans Swing, and Apache Pivot. There is also a JavaFX UI for ImageJ2 developed by Cyril Mongis at the University of Heidelberg [53], as well as integrations of ImageJ into other powerful end-user applications such as KNIME and CellProfiler. See Fig. 4 for a side-by-side illustration of UIs.

**Interoperability.** There is no one-size-fits-all tool for scientific image processing. A diversity of tools benefits users, even more so when they can interoperate. ImageJ 1.x was designed to be run by a single user on a single desktop computer. Many aspects of the program are structured as singletons: one macro interpreter, one WindowManager, one active image, one PlugInClassLoader, one active ROI per image, one set of overlays, one active ROI in the ROI manager, etc. This structure imposes many limitations: for example, multiple macros cannot run concurrently, and it is not possible to operate more than one instance of ImageJ 1.x in the same JVM simultaneously—e.g., on a single web page as applets.

ImageJ2 is structured as an application container, avoiding the static singleton pattern and hence many of ImageJ 1.x’s limitations. Multiple instances of ImageJ2 can run in the same JVM, each with multiple (or no) user interfaces and multiple concurrent operations. Our primary goal is to make each encapsulated component of ImageJ2 usable in other software projects. There are several examples of other projects leaning on this generality to expose SciJava modules in interesting ways: for example, automated conversion to nodes in a KNIME workflow. Continuing efforts are underway at the Laboratory for Optical and Computational Instrumentation (LOCI) and elsewhere to integrate ImageJ with many other software projects, script languages and paradigms (Table 1).

### Reproducibility

In the interest of transparency and reproducibility—especially in the context of open science—the ImageJ2 project strives to be accessible. Ultimately, we want to spur the community to improve ImageJ in a collaborative way, by providing open access to resources. Of course, we recognize the need for responsive, reliable maintainers to coordinate and facilitate contributions. However, with the Internet’s modern software infrastructure, it is now quite feasible to push ImageJ development in a more collaborative and community-driven direction, embracing the “GitHub effect” [54] of worldwide, distributed development.

All ImageJ2 source code is open and publicly available on GitHub [55], and all core components are permissively licensed [56] to avoid any ambiguity over how the code can be used. But visibility alone is not sufficient to keep a project open; each line of code adds complexity, making the project harder to understand and maintain. Modular, encapsulated design, the application of the “Don’t Repeat Yourself (DRY)” concept, and avoidance of overly “clever” code keeps ImageJ2 well-organized and easier to understand. Extensive online documentation [29] and Javadoc [57] provide further insight, while effective unit testing and dedicated tutorial components [58] illustrate concrete use cases. By keeping a clean, well-organized and well-documented codebase, we facilitate community contributions, as well as continued maintenance of ImageJ into the future.

ImageJ2’s open development process provides many benefits over the centralized process of ImageJ 1.x. The use of Maven makes dependency management human readable and enables the use of a “bill of materials” to unambiguously determine which versions of each ImageJ component are compatible. The use of Git has evolved revision control to a new level of documentation, clearly communicating why changes are made and encouraging atomic, easily understood changes. Furthermore, ImageJ2 minimizes the barrier to community contributions via an open issue tracking system [59] and open patch submission process [60].

Finally, it is critical for reproducibility that algorithms produce consistent results over time. We utilize the public Travis continuous integration (CI) infrastructure [61] to run automated regression tests whenever modifications to ImageJ’s source code are published. Such tests help to avoid and detect regression bugs so that core functionality and behavior will continue to work reliably as the program evolves. We especially prioritize test coverage for the crucial base levels of ImageJ: as of this writing, there are approximately 500 tests for SciJava Common, 1200 tests for ImageJ Ops, and 1200 tests for ImageJ 1.x. We also plan to integrate automated test coverage analysis, to measure the percentage of code which is exercised by the tests, which should be straightforward thanks to the project’s use of Maven.

### Usability

The ImageJ community includes both end users—who use ImageJ as an application and want it to “just work”—and software developers—who want to customize and invoke parts of ImageJ as a software library from their own programs. However, these roles are not rigid; many users write scripts and macros to facilitate their image analysis, and many developers also use ImageJ as an application. ImageJ2 includes a powerful Script Editor with many tools to aid users as they transition into the realm of development. This tool removes much of the complexity of traditional software development, allowing users to focus on coding without the added burden of applying compilers, Integrated Development Environments (IDEs), or the command line.

**Fig. 5:**
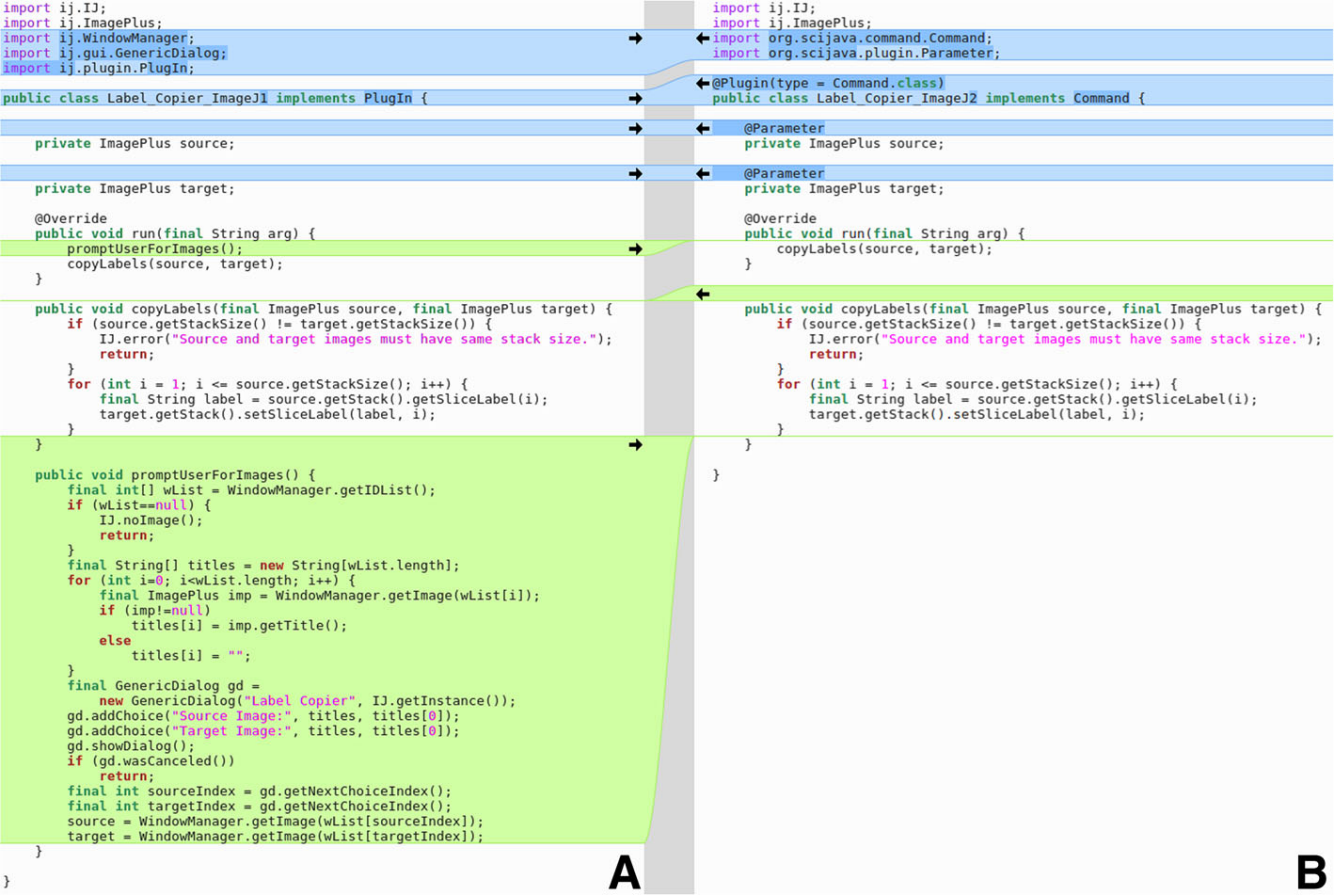
Comparison of pure ImageJ 1.x command with one using SciJava declarative syntax. Panel **a** (left) shows an ImageJ 1.x implementation of a plugin that copies slice labels from one image to another, as chosen by the user. Panel **b** (right) shows the same plugin written using the SciJava declarative command syntax. Changed lines are highlighted in blue, new lines in green. The actual operation (the copyLabels method) is identical, but the routine for selecting which images to process is no longer necessary

In addition, the SciJava parameterized scripting mechanism makes it easier for users to write scripts whose inputs and outputs are declared in a clear and straightforward manner. SciJava parameters reduce the boilerplate code historically needed to define a script’s input values, in some cases by 50% or more (Fig. 5). Leveraging SciJava annotations also frees the plugin from the Java AWT dependencies of ImageJ 1.x’s GenericDialog, allowing the plugin to be used headlessly, in future UIs, and even in other applications.

**Sensible defaults.** A key example of reasonable default behavior is the SciJava conversion framework with its specialized Converter plugin type. Converter plugins define useful type substitutions that would not normally be allowed by the Java type hierarchy. For example, conversion of ImageJ Dataset objects to and from other paradigms (MATLAB arrays, ITK images, etc.) is facilitated by Converter plugins which encapsulate the logic for each conversion case. The framework then uses the converters automatically when modules are executed, so when a user says e.g. “run this ITK algorithm on this dataset I opened” everything “just works” without the user needing to perform an explicit manual conversion. From a software development perspective, this scheme lets ImageJ2 retain the advantages of strong typing while escaping the corresponding shackles that often accompany it.

The ImageJ Ops library provides another illustration of ImageJ2’s sensible defaults structured in layers. While every op in the system is a dynamically callable plugin, the core Ops library also organizes its built-in operations into a centrally accessible collection of type-safe namespaces in a standard Java API explorable from IDEs like Eclipse e.g. via its Content Assist functions. This structure also provides an elegant and easy-to-read syntax for calling ops in script-driven workflows (see Fig. 7 in “Compatibility” below).

**Automatic updates.** In ImageJ 1.x, plugin installation requires users to download a Java ARchive (JAR) or Java class file and place it within the ImageJ plugins folder. Updating an installed plugin essentially requires repeating the manual installation steps, which is both tedious and error-prone. Developers have to manually manage their plugin’s dependencies, which in practice leads to users receiving cryptic error messages when multiple plugins require incompatible component versions. Even worse, some developers then make suboptimal design decisions to work around this difficulty, such as reimplementing functionality already provided by well-tested third party libraries, and/or creating so-called “uber-JARs” which lump together the dependencies into intractable bundles [62].

The ImageJ Updater vastly simplifies this process by informing users automatically when a new plugin version is available and enabling single-click upgrades to the latest version of all components. On the development side, the use of Maven by the ImageJ2 and Fiji projects provides a clear best practice for managing dependencies in a consistent way, which reduces the chance of broken end-user installations.

ImageJ’s support for multiple update sites makes it feasible for community developers and third parties to create their own update sites from which users can pick and choose, without them needing to become a part of the core ImageJ or Fiji distribution. This distributed model of update sites fits in very well with the community-driven aspects of ImageJ, dramatically lowering the barrier for sharing effort. This capability is made even more potent by the Personal Update Sites feature, which lets users link their ImageJ wiki account to their own personal web space. Furthermore, the Updater derives its initial list of available update sites from the “List of update sites” wiki page of the ImageJ website [21]—plugin developers can edit this wiki page in the same way as the rest of the ImageJ website, in order to make their site automatically available to all users of ImageJ. Editing this page is not mandatory, however; users can also manually edit their ImageJ installation’s list of available update sites—e.g., if their organization offers an internally managed update site for plugins specific to their institute.

Although manual plugin installation is still supported in ImageJ2, many organizations have already publicized their own update sites, and thanks to the Updater together with the ImageJ Legacy layer, all of the plugins served from those sites are easily accessible within ImageJ2. This has helped to focus the Fiji project on its original goal of being a curated collection of plugins facilitating scientific image analysis. In addition to Fiji, hundreds of third-party update sites are available, such as LOCI, Broadly Applicable Routines (BAR), BioVoxxel, the Stowers Institute, and the BioImaging and Optics platform of the École Polytechnique Fédérale de Lausanne (EPFL), most of which are served from the centrally managed Personal Update Sites server [63].

#### Performance

ImageJ2 is engineered to accommodate the growing size and complexity of image data. Although performance has been a serious design consideration, we believe in aggressive performance optimization only on an as-needed basis as software components stabilize and mature [64]. By designing a robust framework that allows for specialization at every level, we avoid compromising design for the sake of incremental performance gains, while empowering developers to optimize when necessary. Furthermore, the fact that ImageJ2 maintains 100% backwards compatibility with ImageJ 1.x (see “Compatibility” below) means that existing high-performance image processing approaches continue to work as is, even if they do not benefit from ImageJ2’s architectural improvements.

**Efficiency.** The time performance of ImageJ2 data structures is generally consistent with those of ImageJ 1.x. The core of performance in ImageJ2 hinges on the efficiency of the various ImgLib2 containers. We have run benchmarks comparing the time performance of iteration and access on ImgLib2 image structures with that of ImageJ 1.x images, as well as compared to raw array access [65]. We found that thanks to Java’s Just-In-Time compiler (JIT), ImgLib2 is highly comparable to ImageJ 1.x in these regards (Additional file 1: Figure S2).

When time performance is dominated by the overhead of looping itself, some ImgLib2 container types such as cell images may be measurably slower to iterate and access. However, this loop overhead is generally very small, and for most container types (e.g., array and planar images) the JIT quickly optimizes the code to equal the speed of raw array access. Hence, for non-trivial image processing operations which take significant time to compute per sample, overall time performance converges across all data structures and container types, ImageJ 1.x and ImageJ2 alike.

The advantages of ImgLib2’s type- and container-agnostic algorithm development outweigh any minor differences in time performance, saving developer time and effort via simpler, more maintainable code. Furthermore, ImgLib2’s more comprehensive set of image types (Additional file 1: Table S3) make it easier to optimize for space efficiency. For example, an image sequence recorded using a 12-bit detector requires 16 bits per sample in ImageJ 1.x, whereas ImageJ2 can pack that data without wasted bits using ImgLib2’s uint12 data type, resulting in a 33% increase in space efficiency.

Relatedly, the design of the ImageJ Ops library realizes these same efficiency advantages. End user scripts invoke ops by name and arguments, and the Ops matching algorithm takes care of selecting the implementation optimized for those arguments. This scheme enables image processing algorithms to be written once, then automatically benefit from future performance optimizations without explicit case logic.

**Fig. 6:**
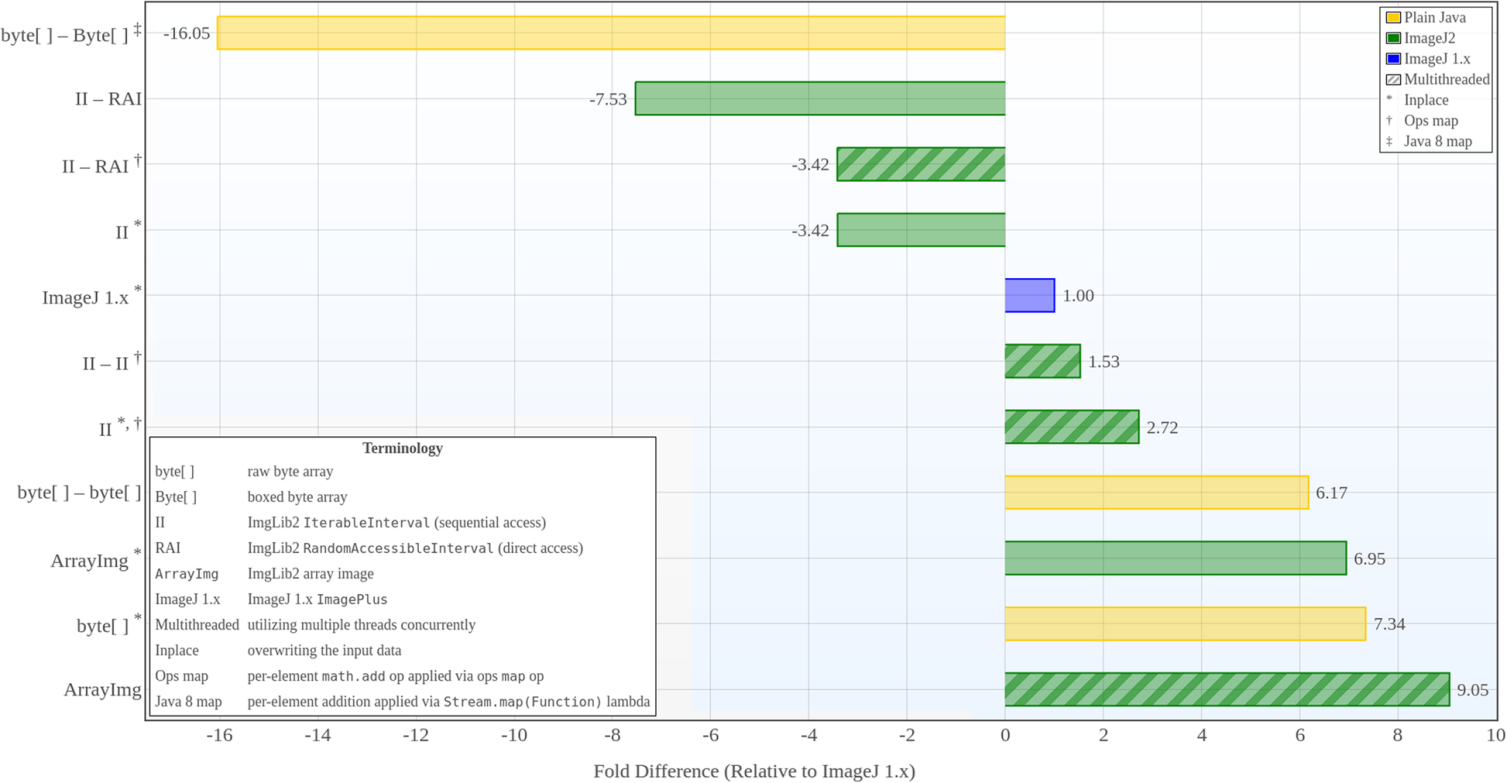
Benchmarks of a simple addition operation with ImageJ Ops and ImageJ 1.x. Time performance comparison of simple addition operations between raw Java array manipulation, various math.add operations of ImageJ Ops, and ImageJ 1.x’s *Process* *⊳* *Math* *⊳* *Add…* command. Benchmarks were run for 20 rounds on randomly generated uint8 noise images dimensioned 15,000×15,000, using the JUnit Benchmarks framework, on a MacBook Pro (Retina, 15-inch, Mid 2015) running macOS Sierra 10.12 with 2.5 GHz Intel Core i7 processor and 16 GB 1600 MHz DDR3 memory. Positive numbers are fold faster, negative numbers are fold slower. The routines which produced these results can be found in the ImageJ Ops test code, in the AddOpBenchmarkTest class of the net.imagej.ops.benchmark package

To validate this approach, we benchmarked the core Ops library’s math.add operations which add a constant value to each element of an image (Fig. 6). As evidenced by the results, the inplace ImageJ 1.x version of this operation (the *Add…* command under Math in the Process menu) performs much better than some generalized op implementations (II source to RAI destination) which work on all image types—but the optimized ops outperform it, with the single-threaded inplace ArrayImg op finishing 7 times faster, and the multithreaded version finishing 9 times faster. While some of this gain is likely due to the expense of ImageJ 1.x’s bounds checking, it is also evident from the results that the optimized ops are comparable in efficiency to operations on raw primitive arrays.

**Scalability.** As discussed in “Functionality” above, ImageJ 1.x is fundamentally limited to XY image planes of less than 2^31^ pixels due to its use of one Java primitive array per plane, and to the size of available computer memory for many of its image processing operations. In contrast, ImageJ2 has been engineered at every level to support scalable image processing using cell images which are cached to and from mass storage on demand. ImageJ2’s careful separation of concerns and enhanced command line parameter handling enable ImageJ to run headless on remote servers, opening up a wide array of possibilities for scalable computation. The SCIFIO library enables direct access into image data samples, so that image data many orders of magnitude larger than available computer memory can be systematically processed on an individual workstation or using a cluster. And thanks to visualization tools like the BigDataViewer plugin [66], which is also built on ImgLib2 cell images, it is now realistic to quickly visualize and explore such massive datasets.

### Compatibility

The ImageJ2 project, by design, enables software developers to use a combination of ImageJ 1.x and ImageJ2 features. Many ImageJ-based tools and plugins continue to rely on ImageJ 1.x data structures, with varying levels of dependence on ImageJ2 and the SciJava framework (Additional file 1: Figure S3). A few examples include:

- **TrackMate** [67], a plugin for object identification and tracking, has been used to achieve a semi-automated workflow for deoxyribonucleic acid (DNA) double-strand break-induced telomere mobility quantitative analysis [68], as well as for *Caenorhabditis-elegans* lineage analysis during light-induced damage, recruitment of NF- *κ*B essential modulator (NEMO) clusters in fibroblasts after Interleukin-1 (IL-1) stimulation, and clathrin-mediated endocytosis analysis in plant cells [67].
- **Massive Multi-view Tracker (MaMuT)**, a tool for the annotation of massive, multi-view data, has been used for reconstruction of the complete cell lineage of an outgrowing thoracic limb of the crustacean *Parhyale hawaiensis*, with single-cell resolution [69].
- **Multiview Reconstruction** [70, 71], a pipeline for registering multi-angle 3D volumes and visualizing them using the BigDataViewer [66], is commonly part of experimental protocols for light sheet fluorescence microscopy, and has been used to analyze zebrafish embryo eye development [72], as well as directional movement of cerebrospinal fluid in zebrafish larvae during developmental neurogenesis [73].
- **Sholl Analysis** [74] and **Simple Neurite Tracer (SNT)** [75], plugins for quantifying traced structures such as neurites, have been used to analyze dendritic morphology of the amygdala and hippocampus in conventionally-colonized versus germ-free mice [76], morphologies of retinal ganglion cells from neural retina on poly (lactic-co-glycolic acid) (PLGA) scaffold [77], as well as dendritic complexity and arborization in absence of *α*2-chimaerin, a key regulator of Rac1-dependent signaling [78].

**Fig. 7:**
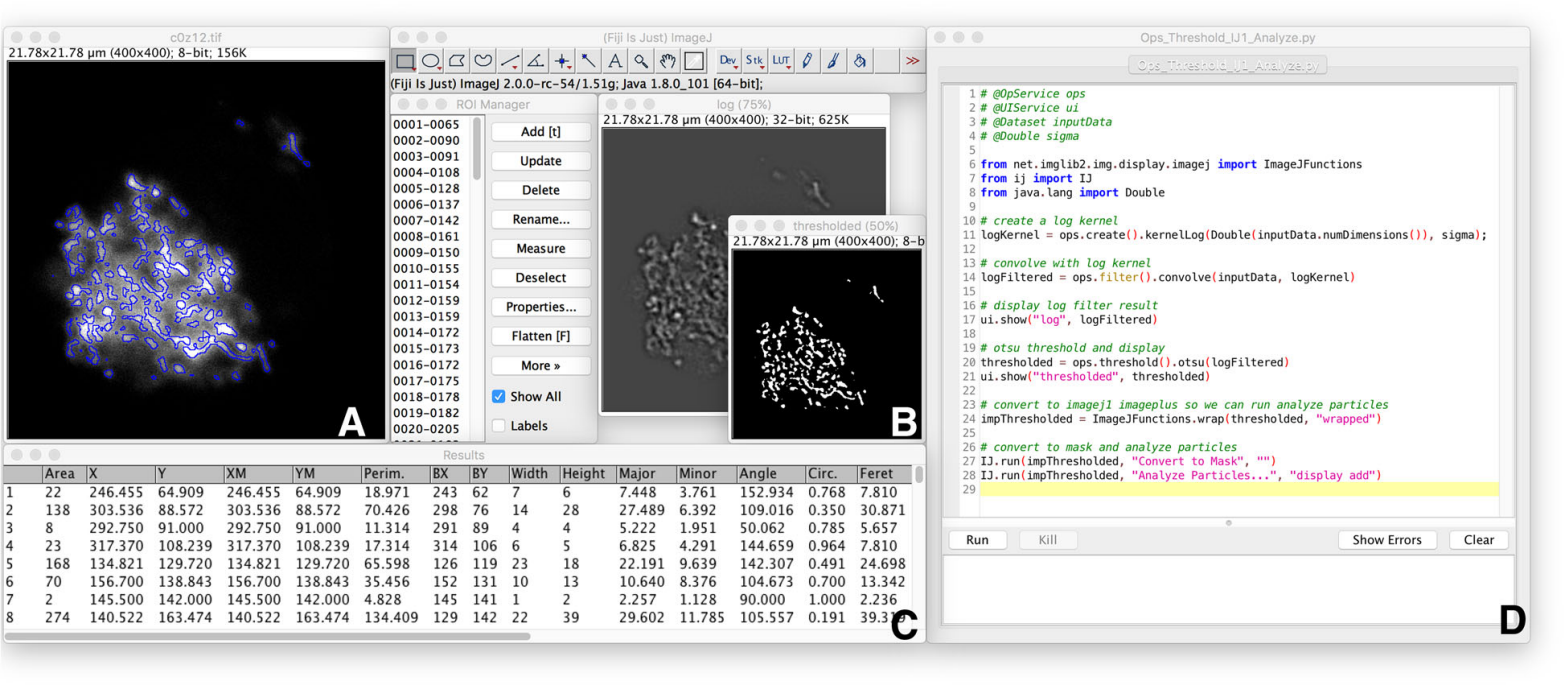
A mixed-world ImageJ 1.x + ImageJ2 script. This example Python script (panel **d**) uses ImageJ Ops to preprocess a confocal image and perform an automatic thresholding (panel **b**). ImageJ 1.x’s Analyze Particles routine is then called to isolate (panel **a**) and measure (panel **c**) foreground objects. Script contributed by Brian Northan, True North Intelligent Algorithms LLC. This script is available within ImageJ as a sample from the Tutorials submenu of the Script Editor’s Templates menu

It is thanks to the ImageJ Legacy and IJ1-patcher components that the community can blend the usage of ImageJ 1.x and ImageJ2 functionality, cherry-picking the best from each world to accomplish their image analysis tasks. For example, parameterized ImageJ commands and scripts may continue to use ImageJ 1.x data structures and plugins as needed, while taking advantage of ImageJ2 functionality as appropriate (Fig. 7), and declaring and populating input values with less boilerplate code (see Fig. 5 in “Usability” above).

As ImageJ2 continues to mature, usage of ImageJ 1.x functionality will be increasingly replaced with more powerful ImageJ2 equivalents: image processing algorithms built on ImageJ Ops, data format plugins built on SCIFIO, block-based cell images using ImgLib2, N-dimensional ROIs, metadata-rich images, nonlinear registration transforms, etc. However, this process is both lengthy and necessarily incomplete: migrating ImageJ 1.x core functionality alone is a years-long process as the ImageJ2 APIs continue to evolve, mature and stabilize—and there are countless other useful plugins and scripts in the wild, some of which will never be updated to the new APIs. Meanwhile, development of ImageJ 1.x also progresses, with users continuing to request bug fixes and new features within its intended scope. As such, the importance of a robust transitional strategy for migrating from ImageJ 1.x to ImageJ2 cannot be overstated.

Although the development of ImageJ2 has necessitated reimplementation of ImageJ 1.x functionality, maintaining backwards compatibility with ImageJ 1.x will remain a fundamental goal. Abandoning or ignoring ImageJ 1.x would have been a significant disservice to the community, causing a rift to the detriment of all parties. Our efforts to enable incremental migration from ImageJ 1.x to ImageJ2 allow the two projects to continue developing in tandem, with new features in each reaching a unified ImageJ community.

### Community

Ultimately, the goal of ImageJ is to enable scientific collaboration and achievement, which requires community management as much as code management. ImageJ 1.x leverages open-source code, a public web site and a mailing list to support discussion and contributions from people across the globe. However, it follows a centralized “cathedral” development model, rather than a community-driven “bazaar” style model [79], with its primary resources and scalability fundamentally limited by a single “gatekeeper.” For ImageJ’s continued success and growth, it is critical to renew its focus on partnership and communication with the community [80].

**Online resources.** The centrally organized documentation of ImageJ2 takes the form of a collaborative wiki [29] with over 800 articles: a “world-writable” location for both users and developers to learn about and contribute to ImageJ. The wiki is complemented by the ImageJ Forum [81], a powerful, friendly and universally accessible discussion channel driven by the excellent Discourse software, which is engineered to encourage civil interaction [82]. Finally, ImageJ’s source code and issue tracking via GitHub completes the community-centric approach for managing and discussing changes and improvements.

These resources together enable project developers to clearly communicate the expectations and norms surrounding plugin development, contribution, maintenance and support, empowering users to easily see who is responsible for each plugin as well as its support and development status [83], outstanding bugs [59] and future plans [84]. This is a critical service for the community: it is not enough to provide a convenient way for people to publish, share and consume extensions—we have learned from experience that there must be a social framework in place for managing and understanding the software development lifecycle of the myriad community efforts.

### Future directions

ImageJ is more than a single application: it is a living ecosystem of scientific exchange. As acquisition technology continues to advance, there will always be a need for new development and maintenance within ImageJ. There are still key technical tasks remaining for ImageJ2 to achieve stability, as well as new directions made possible by the ImageJ2 platform which we are excited to explore:

- Finalize the ImageJ Common data model to support extensible attachment of metadata, including spatial metadata, that respond robustly to image processing operations such as transformation.
- Extend ImgLib2’s N-dimensional ROI interfaces to cover all needed cases, including all ROI types supported by ImageJ 1.x, all ROI types supported by OMERO, and any additional ROI types available in other image-oriented software packages for which integration with ImageJ is pursued.
- Update the core SciJava I/O mechanism to be plugin-driven for improved extensibility of data source location.
- Generalize SCIFIO’s planar model to operate on arbitrary “blocks” at a fundamental level.
- Retire the custom C-based ImageJ desktop application launcher, migrating to the industry-standard application bundling of JavaFX.
- Complete our ongoing effort to automate the documentation regarding development and maintenance responsibility for every core component of the ImageJ ecosystem, including Fiji components [83].
- Develop our ImageJ-based REST image server prototype toward production use, providing a common language- and implementation-independent API.
- Implement a web UI built on the REST image server.
- Improve the ImageJ Updater user interface to be more user friendly, so that users can more easily cherry-pick extensions of interest from each update site.
- Expand the list of built-in ImageJ Ops with additional image processing and analysis routines, including Deep Learning approaches [85] and novel algorithms from computer vision and statistics.
- Continue building bridges between ImageJ and other image processing frameworks such as OpenCV [86] and scikit-image [87].
- Integrate support for cloud computing frameworks such as Apache Spark [46] running on platforms such as Amazon Web Services [88].
- Continue supporting community requests for bug fixes, new features and image analysis advice.
- Continue migrating ImageJ resources into the main ImageJ wiki website, including the ImageJ User Guide [89], ImageJ 1.x documentation [90] and Luxembourg Institute of Science and Technology (LIST)’s ImageJ Information and Documentation Portal [91].
- Redesign ImageJ’s bug submission system such that users can automatically submit an issue report to the correct location online whenever something goes wrong in the software.
- Continue listening to, and working with, the user and developer community.

A detailed breakdown and discussion of each specific issue can be found on GitHub, searchable from the unified ImageJ Search portal [92].

## Conclusions

Based on feedback from the existing ImageJ community, we have over the last several years been designing and implementing ImageJ2, a radically improved application that employs best practices and proven software engineering approaches. ImageJ2 directly addresses two major needs, supporting applications where: 1) the underlying ImageJ data engine was not sufficient to analyze modern datasets; and 2) the lack of an underlying robust software design impeded the addition of new functionality. This overhaul of ImageJ transforms it into not only a powerful and flexible image processing and analysis application in its own right, but also a framework for interoperability between a plethora of external image visualization and analysis programs. ImageJ2 strengthens ImageJ’s utility as a platform for scientific image analysis by: 1) generalizing the ImageJ data model; 2) introducing a robust architecture instrumental in building bridges across a range of other image processing tools; 3) remaining open source and cross-platform with permissive licensing, enabling continued widespread adoption and extension; 4) building on the huge collection of existing ImageJ plugins while enabling the creation of new plugins with more powerful features; and 5) leveraging a correspondingly large and diverse community to foster a collaborative and interdisciplinary project that facilitates the collective advancement of science.

## Availability and requirements

**Project name:** ImageJ

**Project home page:** https://imagej.net/

**Archived version:** net.imagej:imagej:2.0.0-rc-55

**Operating system(s):** Platform independent

**Programming language:** Java

**Other requirements:** Java 8 or higher

**License:** Simplified (2-clause) Berkeley Software Distribution (BSD)

**Any restrictions to use by non-academics:** None

All data generated or analyzed during this study are included in this published article.

## Additional file

Additional file 1 Figures and illustrations. **Figure S1**: Module execution in different contexts. When running a parameterized script (panel B) from the ImageJ user interface (panel A), a pop-up dialog box (panel C) enables the user to enter the name and age values; when running the script headless from the command line (panel D), input values are passed as arguments and output values echoed to the standard output stream. **Figure S2**: Comparison of time performance across ImageJ 1.x and ImgLib2 data structures. For ten iterations, we ran a “cheap” per-pixel operation and an “expensive” operation on a 25 Mpx image stored in the ImageJ 1.x container, various ImgLib2 containers, and raw byte arrays. Panel A (left) shows the time (ms) it took to complete a “cheap” operations versus the loop iteration for each container. Panel B (right) shows the same information but for the time (ms) it took to complete the expensive operation. **Figure S3**: Sample ImageJ plugin usage of ImageJ 1.x and ImageJ2. This plot displays a select few ImageJ plugins in varying stages of transition, from ImageJ 1.x to ImageJ2, as of 11 Aug 2017 2:35 PM CDT. The ratio of ImageJ 1.x to ImageJ2 usage was computed by counting the number of imports each plugin uses from relevant Java packages: "ImageJ 1.x plugins" is ij.plugin.*, "ImageJ 1.x data structures" is ij.* excluding the plugin subpackage, "SciJava framework" is org.scijava.*, and "ImageJ2 data structures" is net.imagej.* and net.imglib2.*. References for plugins shown: TrackMate [67], MaMuT [150], Multiview Reconstruction [70, 71], MotherMachine Analyzer (MoMA) [151, 152], Sholl Analysis [74], Kymograph Builder [153], Z-Spacing Correction [154], Trainable Weka Segmentation [155], Pendent Drop [156], SciView [157], BigDataViewer [66], Image Stitching [158], Coloc 2 [159], MorphoLibJ [100]. **Table S1**: Built-in SciJava input widgets. **Table S2**: Kinds and arities of special ops. **Table S3**: Image types supported by ImageJ. (ZIP 549 kb)

## Abbreviations

3D: Three-dimensional
Alida: Advanced library for integrated development of data analysis applications
API: Application programming interface
AWT: Abstract windowing toolkit
BAR: Broadly applicable routines
BeakerX: Beaker extensions for Jupyter
BisQue: Bio-Image semantic query user environment
BMC: BioMed Central
BSD: Berkeley software distribution
CI: Continuous integration
CPU: Central processing unit
CUDA: Compute unified device architecture
DI: Dependency injection
DNA: Deoxyribonucleic acid
DRY: Don’t repeat yourself
EPFL: École Polytechnique Fédérale de Lausanne
Fiji: Fiji is just ImageJ
GPU: Graphics processing unit
GTK: GIMP ToolKit
HTTP: Hypertext transfer protocol
I/O: Input/Output
IDE: Integrated development environment
IL-1: Interleukin-1
IoC: Inversion of control
ITK: Insight ToolKit
JAR: Java ARchive
JIT: Just-In-Time compiler
JVM: Java virtual machine
KNIME: KoNstanz information MinEr
KNIP: KNIME image processing
L&F: Look & feel
LIST: Luxembourg institute of science and technology
LOCI: Laboratory for optical and computational instrumentation
LUT: Color lookup table
macOS: Macintosh operating system
MaMuT: Massive multi-view tracker
MATLAB: MATrix LABoratory
MiToBo: Microscopy image analysis ToolBox
MoMA: MotherMachine analyzer
NEMO: NF- *κ B essential modulator*
NIGMS: National institute of general medical sciences
NIH: National institutes of health
OME: Open microscopy environment
OMERO: OME remote objects
OpenCL: Open computing language
OpenCV: Open source computer vision library
PLGA: Poly (lactic-co-glycolic acid)
RAM: Random-Access memory
REST: REpresentational state transfer
RGB: Red+Green+Blue color model
ROI: Region of interest
SCIFIO: SCientific image format input and output
SCP: Secure CoPy
SFTP: Secure file transfer protocol
SNT: Simple neurite tracer
SSH: Secure SHell
SWT: Standard widget toolkit
UI: User interface
URI: Uniform resource identifier
URL: Uniform resource locator
WebDAV: Web distributed authoring and versioning
XML: eXtensible Markup Language
