# Supplementary figures and images for: ImageJ2: ImageJ for the next generation of scientific image data
Source: BMC Bioinformatics. 2017 Nov 29;18:529. doi: 10.1186/s12859-017-1934-z (PMC5708080; doi:10.1186/s12859-017-1934-z)

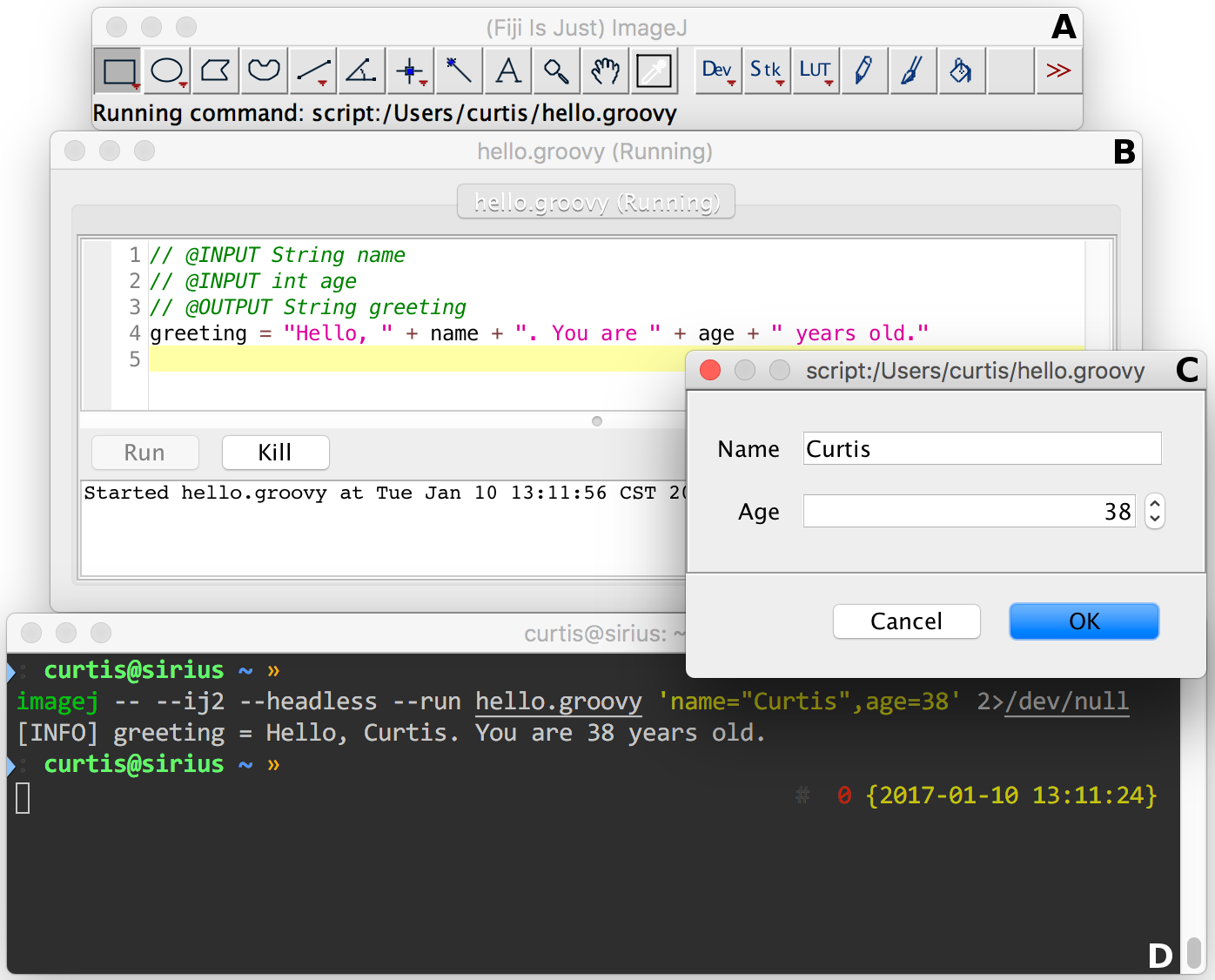

Supplement: Additional file 1 — Figures and illustrations. Figure S1: Module execution in different contexts. When running a parameterized script (panel B) from the ImageJ user interface (panel A), a pop-up dialog box (panel C) enables the user to enter the name and age values; when running the script headless from the command line (panel D), input values are passed as arguments and output values echoed to the standard output stream. Figure S2: Comparison of time performance across ImageJ 1.x and ImgLib2 data structures. For ten iterations, we ran a “cheap” per-pixel operation and an “expensive” operation on a 25 Mpx image stored in the ImageJ 1.x container, various ImgLib2 containers, and raw byte arrays. Panel A (left) shows the time (ms) it took to complete a “cheap” operations versus the loop iteration for each container. Panel B (right) shows the same information but for the time (ms) it took to complete the expensive operation. Figure S3: Sample ImageJ plugin usage of ImageJ 1.x and ImageJ2. This plot displays a select few ImageJ plugins in varying stages of transition, from ImageJ 1.x to ImageJ2, as of 11 Aug 2017 2:35 PM CDT. The ratio of ImageJ 1.x to ImageJ2 usage was computed by counting the number of imports each plugin uses from relevant Java packages: "ImageJ 1.x plugins" is ij.plugin.*, "ImageJ 1.x data structures" is ij.* excluding the plugin subpackage, "SciJava framework" is org.scijava.*, and "ImageJ2 data structures" is net.imagej.* and net.imglib2.*. References for plugins shown: TrackMate [67], MaMuT [150], Multiview Reconstruction [70, 71], MotherMachine Analyzer (MoMA) [151, 152], Sholl Analysis [74], Kymograph Builder [153], Z-Spacing Correction [154], Trainable Weka Segmentation [155], Pendent Drop [156], SciView [157], BigDataViewer [66], Image Stitching [158], Coloc 2 [159], MorphoLibJ [100]. Table S1: Built-in SciJava input widgets. Table S2: Kinds and arities of special ops. Table S3: Image types supported by ImageJ. (ZIP 549 kb) [file 12859_2017_1934_MOESM1_ESM.zip › Supplement/figure-s.1.png]

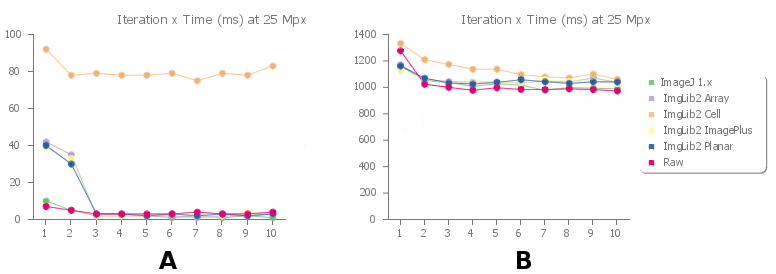

Supplement: Additional file 1 — Figures and illustrations. Figure S1: Module execution in different contexts. When running a parameterized script (panel B) from the ImageJ user interface (panel A), a pop-up dialog box (panel C) enables the user to enter the name and age values; when running the script headless from the command line (panel D), input values are passed as arguments and output values echoed to the standard output stream. Figure S2: Comparison of time performance across ImageJ 1.x and ImgLib2 data structures. For ten iterations, we ran a “cheap” per-pixel operation and an “expensive” operation on a 25 Mpx image stored in the ImageJ 1.x container, various ImgLib2 containers, and raw byte arrays. Panel A (left) shows the time (ms) it took to complete a “cheap” operations versus the loop iteration for each container. Panel B (right) shows the same information but for the time (ms) it took to complete the expensive operation. Figure S3: Sample ImageJ plugin usage of ImageJ 1.x and ImageJ2. This plot displays a select few ImageJ plugins in varying stages of transition, from ImageJ 1.x to ImageJ2, as of 11 Aug 2017 2:35 PM CDT. The ratio of ImageJ 1.x to ImageJ2 usage was computed by counting the number of imports each plugin uses from relevant Java packages: "ImageJ 1.x plugins" is ij.plugin.*, "ImageJ 1.x data structures" is ij.* excluding the plugin subpackage, "SciJava framework" is org.scijava.*, and "ImageJ2 data structures" is net.imagej.* and net.imglib2.*. References for plugins shown: TrackMate [67], MaMuT [150], Multiview Reconstruction [70, 71], MotherMachine Analyzer (MoMA) [151, 152], Sholl Analysis [74], Kymograph Builder [153], Z-Spacing Correction [154], Trainable Weka Segmentation [155], Pendent Drop [156], SciView [157], BigDataViewer [66], Image Stitching [158], Coloc 2 [159], MorphoLibJ [100]. Table S1: Built-in SciJava input widgets. Table S2: Kinds and arities of special ops. Table S3: Image types supported by ImageJ. (ZIP 549 kb) [file 12859_2017_1934_MOESM1_ESM.zip › Supplement/figure-s.2.png]

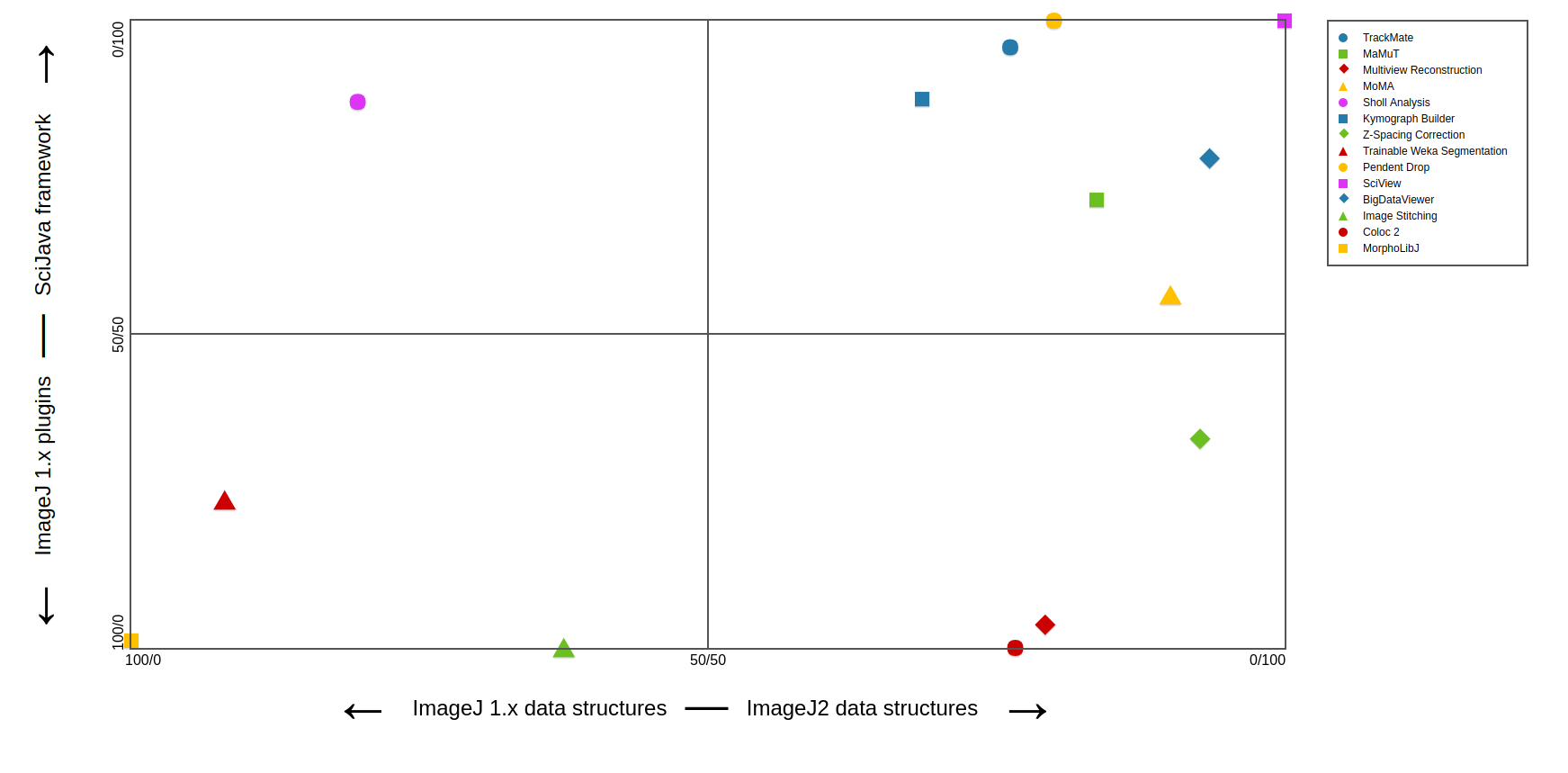

Supplement: Additional file 1 — Figures and illustrations. Figure S1: Module execution in different contexts. When running a parameterized script (panel B) from the ImageJ user interface (panel A), a pop-up dialog box (panel C) enables the user to enter the name and age values; when running the script headless from the command line (panel D), input values are passed as arguments and output values echoed to the standard output stream. Figure S2: Comparison of time performance across ImageJ 1.x and ImgLib2 data structures. For ten iterations, we ran a “cheap” per-pixel operation and an “expensive” operation on a 25 Mpx image stored in the ImageJ 1.x container, various ImgLib2 containers, and raw byte arrays. Panel A (left) shows the time (ms) it took to complete a “cheap” operations versus the loop iteration for each container. Panel B (right) shows the same information but for the time (ms) it took to complete the expensive operation. Figure S3: Sample ImageJ plugin usage of ImageJ 1.x and ImageJ2. This plot displays a select few ImageJ plugins in varying stages of transition, from ImageJ 1.x to ImageJ2, as of 11 Aug 2017 2:35 PM CDT. The ratio of ImageJ 1.x to ImageJ2 usage was computed by counting the number of imports each plugin uses from relevant Java packages: "ImageJ 1.x plugins" is ij.plugin.*, "ImageJ 1.x data structures" is ij.* excluding the plugin subpackage, "SciJava framework" is org.scijava.*, and "ImageJ2 data structures" is net.imagej.* and net.imglib2.*. References for plugins shown: TrackMate [67], MaMuT [150], Multiview Reconstruction [70, 71], MotherMachine Analyzer (MoMA) [151, 152], Sholl Analysis [74], Kymograph Builder [153], Z-Spacing Correction [154], Trainable Weka Segmentation [155], Pendent Drop [156], SciView [157], BigDataViewer [66], Image Stitching [158], Coloc 2 [159], MorphoLibJ [100]. Table S1: Built-in SciJava input widgets. Table S2: Kinds and arities of special ops. Table S3: Image types supported by ImageJ. (ZIP 549 kb) [file 12859_2017_1934_MOESM1_ESM.zip › Supplement/figure-s.3.png]

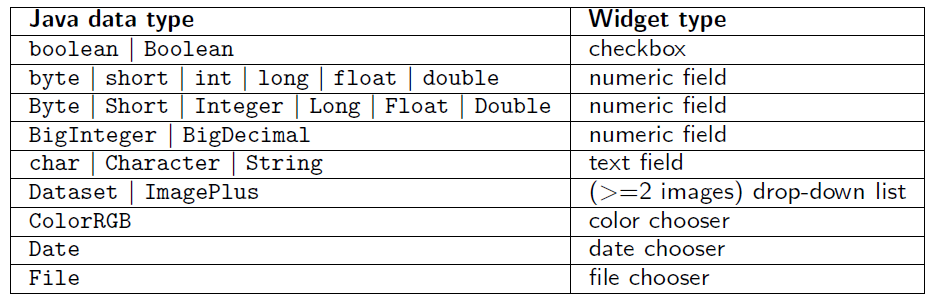

Supplement: Additional file 1 — Figures and illustrations. Figure S1: Module execution in different contexts. When running a parameterized script (panel B) from the ImageJ user interface (panel A), a pop-up dialog box (panel C) enables the user to enter the name and age values; when running the script headless from the command line (panel D), input values are passed as arguments and output values echoed to the standard output stream. Figure S2: Comparison of time performance across ImageJ 1.x and ImgLib2 data structures. For ten iterations, we ran a “cheap” per-pixel operation and an “expensive” operation on a 25 Mpx image stored in the ImageJ 1.x container, various ImgLib2 containers, and raw byte arrays. Panel A (left) shows the time (ms) it took to complete a “cheap” operations versus the loop iteration for each container. Panel B (right) shows the same information but for the time (ms) it took to complete the expensive operation. Figure S3: Sample ImageJ plugin usage of ImageJ 1.x and ImageJ2. This plot displays a select few ImageJ plugins in varying stages of transition, from ImageJ 1.x to ImageJ2, as of 11 Aug 2017 2:35 PM CDT. The ratio of ImageJ 1.x to ImageJ2 usage was computed by counting the number of imports each plugin uses from relevant Java packages: "ImageJ 1.x plugins" is ij.plugin.*, "ImageJ 1.x data structures" is ij.* excluding the plugin subpackage, "SciJava framework" is org.scijava.*, and "ImageJ2 data structures" is net.imagej.* and net.imglib2.*. References for plugins shown: TrackMate [67], MaMuT [150], Multiview Reconstruction [70, 71], MotherMachine Analyzer (MoMA) [151, 152], Sholl Analysis [74], Kymograph Builder [153], Z-Spacing Correction [154], Trainable Weka Segmentation [155], Pendent Drop [156], SciView [157], BigDataViewer [66], Image Stitching [158], Coloc 2 [159], MorphoLibJ [100]. Table S1: Built-in SciJava input widgets. Table S2: Kinds and arities of special ops. Table S3: Image types supported by ImageJ. (ZIP 549 kb) [file 12859_2017_1934_MOESM1_ESM.zip › Supplement/Table S1.PNG]

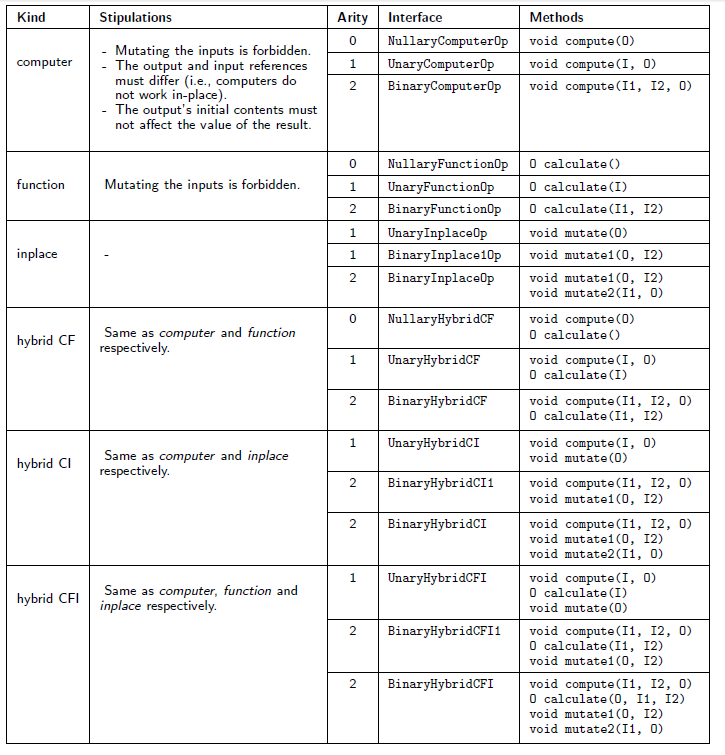

Supplement: Additional file 1 — Figures and illustrations. Figure S1: Module execution in different contexts. When running a parameterized script (panel B) from the ImageJ user interface (panel A), a pop-up dialog box (panel C) enables the user to enter the name and age values; when running the script headless from the command line (panel D), input values are passed as arguments and output values echoed to the standard output stream. Figure S2: Comparison of time performance across ImageJ 1.x and ImgLib2 data structures. For ten iterations, we ran a “cheap” per-pixel operation and an “expensive” operation on a 25 Mpx image stored in the ImageJ 1.x container, various ImgLib2 containers, and raw byte arrays. Panel A (left) shows the time (ms) it took to complete a “cheap” operations versus the loop iteration for each container. Panel B (right) shows the same information but for the time (ms) it took to complete the expensive operation. Figure S3: Sample ImageJ plugin usage of ImageJ 1.x and ImageJ2. This plot displays a select few ImageJ plugins in varying stages of transition, from ImageJ 1.x to ImageJ2, as of 11 Aug 2017 2:35 PM CDT. The ratio of ImageJ 1.x to ImageJ2 usage was computed by counting the number of imports each plugin uses from relevant Java packages: "ImageJ 1.x plugins" is ij.plugin.*, "ImageJ 1.x data structures" is ij.* excluding the plugin subpackage, "SciJava framework" is org.scijava.*, and "ImageJ2 data structures" is net.imagej.* and net.imglib2.*. References for plugins shown: TrackMate [67], MaMuT [150], Multiview Reconstruction [70, 71], MotherMachine Analyzer (MoMA) [151, 152], Sholl Analysis [74], Kymograph Builder [153], Z-Spacing Correction [154], Trainable Weka Segmentation [155], Pendent Drop [156], SciView [157], BigDataViewer [66], Image Stitching [158], Coloc 2 [159], MorphoLibJ [100]. Table S1: Built-in SciJava input widgets. Table S2: Kinds and arities of special ops. Table S3: Image types supported by ImageJ. (ZIP 549 kb) [file 12859_2017_1934_MOESM1_ESM.zip › Supplement/Table S2.PNG]

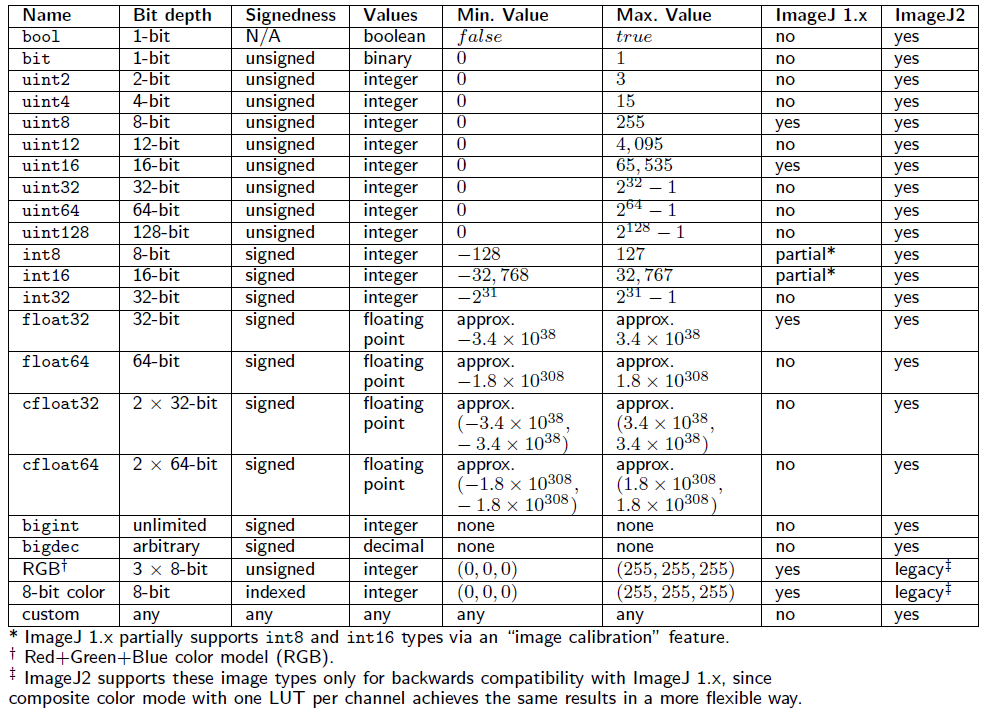

Supplement: Additional file 1 — Figures and illustrations. Figure S1: Module execution in different contexts. When running a parameterized script (panel B) from the ImageJ user interface (panel A), a pop-up dialog box (panel C) enables the user to enter the name and age values; when running the script headless from the command line (panel D), input values are passed as arguments and output values echoed to the standard output stream. Figure S2: Comparison of time performance across ImageJ 1.x and ImgLib2 data structures. For ten iterations, we ran a “cheap” per-pixel operation and an “expensive” operation on a 25 Mpx image stored in the ImageJ 1.x container, various ImgLib2 containers, and raw byte arrays. Panel A (left) shows the time (ms) it took to complete a “cheap” operations versus the loop iteration for each container. Panel B (right) shows the same information but for the time (ms) it took to complete the expensive operation. Figure S3: Sample ImageJ plugin usage of ImageJ 1.x and ImageJ2. This plot displays a select few ImageJ plugins in varying stages of transition, from ImageJ 1.x to ImageJ2, as of 11 Aug 2017 2:35 PM CDT. The ratio of ImageJ 1.x to ImageJ2 usage was computed by counting the number of imports each plugin uses from relevant Java packages: "ImageJ 1.x plugins" is ij.plugin.*, "ImageJ 1.x data structures" is ij.* excluding the plugin subpackage, "SciJava framework" is org.scijava.*, and "ImageJ2 data structures" is net.imagej.* and net.imglib2.*. References for plugins shown: TrackMate [67], MaMuT [150], Multiview Reconstruction [70, 71], MotherMachine Analyzer (MoMA) [151, 152], Sholl Analysis [74], Kymograph Builder [153], Z-Spacing Correction [154], Trainable Weka Segmentation [155], Pendent Drop [156], SciView [157], BigDataViewer [66], Image Stitching [158], Coloc 2 [159], MorphoLibJ [100]. Table S1: Built-in SciJava input widgets. Table S2: Kinds and arities of special ops. Table S3: Image types supported by ImageJ. (ZIP 549 kb) [file 12859_2017_1934_MOESM1_ESM.zip › Supplement/Table S3.PNG]
